# Hierarchical spectral clustering reveals brain size and shape changes in asymptomatic carriers of *C9orf72*

**DOI:** 10.1093/braincomms/fcac182

**Published:** 2022-07-18

**Authors:** Rose Bruffaerts, Dorothy Gors, Alicia Bárcenas Gallardo, Mathieu Vandenbulcke, Philip Van Damme, Paul Suetens, John C van Swieten, Barbara Borroni, Raquel Sanchez-Valle, Fermin Moreno, Robert Laforce, Caroline Graff, Matthis Synofzik, Daniela Galimberti, James B Rowe, Mario Masellis, Maria Carmela Tartaglia, Elizabeth Finger, Alexandre de Mendonça, Fabrizio Tagliavini, Chris R Butler, Isabel Santana, Alexander Gerhard, Simon Ducharme, Johannes Levin, Adrian Danek, Markus Otto, Jonathan D Rohrer, Patrick Dupont, Peter Claes, Rik Vandenberghe, Genetic Frontotemporal dementia Initiative (GENFI), Sónia Afonso, Maria Rosario Almeida, Sarah Anderl-Straub, Christin Andersson, Anna Antonell, Silvana Archetti, Andrea Arighi, Mircea Balasa, Myriam Barandiaran, Nuria Bargalló, Robart Bartha, Benjamin Bender, Alberto Benussi, Sandra Black, Martina Bocchetta, Sergi Borrego-Ecija, Jose Bras, Marta Canada, Valentina Cantoni, Paola Caroppo, David Cash, Miguel Castelo-Branco, Rhian Convery, Thomas Cope, Giuseppe Di Fede, Alina Díez, Diana Duro, Chiara Fenoglio, Catarina B Ferreira, Nick Fox, Morris Freedman, Giorgio Fumagalli, Alazne Gabilondo, Roberto Gasparotti, Serge Gauthier, Stefano Gazzina, Giorgio Giaccone, Ana Gorostidi, Caroline Greaves, Rita Guerreiro, Carolin Heller, Tobias Hoegen, Begoña Indakoetxea, Vesna Jelic, Lize Jiskoot, Hans-Otto Karnath, Ron Keren, Tobias Langheinrich, Maria João Leitão, Albert Lladó, Sandra Loosli, Carolina Maruta, Simon Mead, Lieke Meeter, Gabriel Miltenberger, Rick van Minkelen, Sara Mitchell, Katrina Moore, Jennifer Nicholas, Linn Öijerstedt, Jaume Olives, Sebastien Ourselin, Alessandro Padovani, Jessica Panman, Janne M Papma, Georgia Peakman, Yolande Pijnenburg, Enrico Premi, Sara Prioni, Catharina Prix, Rosa Rademakers, Veronica Redaelli, Tim Rittman, Ekaterina Rogaeva, Pedro Rosa-Neto, Giacomina Rossi, Mar tin Rossor, Beatriz Santiago, Elio Scarpini, Sonja Schönecker, Elisa Semler, Rachelle Shafei, Christen Shoesmith, Miguel Tábuas-Pereira, Mikel Tainta, Ricardo Taipa, David Tang-Wai, David L Thomas, Paul Thompson, Hakan Thonberg, Carolyn Timberlake, Pietro Tiraboschi, Emily Todd, Michele Veldsman, Ana Verdelho, Jorge Villanua, Jason Warren, Carlo Wilke, Ione Woollacott, Elisabeth Wlasich, Henrik Zetterberg, Miren Zulaica

**Affiliations:** Laboratory for Cognitive Neurology, Department of Neurosciences, Experimental Neurology, and Leuven Brain Institute (LBI), KU Leuven, Leuven 3000, Belgium; Computational Neurology, Department of Biomedical Sciences, University of Antwerp, Antwerp 2610, Belgium; Biomedical Research Institute, Hasselt University, Hasselt 3590, Belgium; Department of Electrical Engineering, ESAT/PSI, KU Leuven, Leuven 3000, Belgium; Medical Imaging Research Center, KU Leuven, Leuven 3000, Belgium; Medical Imaging Research Center, KU Leuven, Leuven 3000, Belgium; Psychiatry Department, University Hospitals Leuven, Leuven 3000, Belgium; Department of Neurosciences, KU Leuven—University of Leuven, Experimental Neurology, and Leuven Brain Institute (LBI), Leuven 3000, Belgium; Laboratory of Neurobiology, VIB, Center for Brain & Disease Research, Leuven 3000, Belgium; Department of Electrical Engineering, ESAT/PSI, KU Leuven, Leuven 3000, Belgium; Medical Imaging Research Center, KU Leuven, Leuven 3000, Belgium; Department of Neurology, Erasmus Medical Centre, Rotterdam 3015, Netherlands; Centre for Neurodegenerative Disorders, Department of Clinical and Experimental Sciences, University of Brescia, Brescia 25121, Italy; Alzheimer’s disease and Other Cognitive Disorders Unit, Neurology Service, Hospital Clinic, Institut d’Investigacions Biomediques August Pi I Sunyer, University of Barcelona, Barcelona 08036, Spain; Cognitive Disorders Unit, Department of Neurology, Donostia University Hospital, San Sebastian, Gipuzkoa 20014, Spain; Clinique Interdisciplinaire de Mémoire, Département des Sciences Neurologiques, CHU de Québec, and Faculté de Médecine, Université Laval, QC G1Z 1J4, Canada; Center for Alzheimer Research, Division of Neurogeriatrics, Department of Neurobiology, Care Sciences and Society, Bioclinicum, Karolinska Institutet, Solna 17176, Sweden; Department of Neurodegenerative Diseases, Hertie-Institute for Clinical Brain Research and Center of Neurology, University of Tübingen, Tübingen 72076, Germany; Fondazione IRCCS Ospedale Policlinico, Neurodegenerative Diseases Unit, Milan 20122, Italy; Dipartimento di Scienze Biomediche, Chirurgiche e Odontoiatriche, University of Milan, Milan 20122, Italy; Department of Clinical Neurosciences, University of Cambridge, Cambridge CB2 0SZ, UK; Sunnybrook Health Sciences Centre, Sunnybrook Research Institute, University of Toronto, Toronto M4N 3M5, Canada; Tanz Centre for Research in Neurodegenerative Diseases, University of Toronto, Toronto M4N 3M5, Canada; Department of Clinical Neurological Sciences, University of Western Ontario, London, Ontario N6A 3K7, Canada; Faculty of Medicine, University of Lisbon, Lisbon 1649-028, Portugal; Fondazione IRCCS Istituto Neurologico Carlo Besta, Neurodegenerative Diseases Unit, Milano 20133, Italy; Nuffield Department of Clinical Neurosciences, Medical Sciences Division, University of Oxford, Oxford OX3 9DU, UK; University Hospital of Coimbra (HUC), Neurology Service, Faculty of Medicine, University of Coimbra, Coimbra 3004, Portugal; Division of Neuroscience and Experimental Psychology, Wolfson Molecular Imaging Centre, University of Manchester, Manchester M20 3LJ, UK; Department of Geriatric Medicine, Center for Translational Neuro- and Behavioral Sciences, University Medicine Essen, Essen 45147, Germany; Department of Nuclear Medicine, Center for Translational Neuro- and Behavioral Sciences, University Medicine Essen, Essen 45147, Germany; Department of Psychiatry, McGill University Health Centre, McGill University, Montreal, Quebec 3801, Canada; McConnell Brain Imaging Centre, Montreal Neurological Institute, Department of Neurology & Neurosurgery, McGill University, Montreal 3801, Canada; Neurologische Klinik, Ludwig-Maximilians-Universität München, Munich 81377, Germany; Neurologische Klinik, Ludwig-Maximilians-Universität München, Munich 81377, Germany; Department of Neurology, University of Ulm, Ulm 89081, Germany; Department of Neurodegenerative Disease, Dementia Research Centre, UCL Institute of Neurology, Queen Square, London WC1N 3BG, UK; Laboratory for Cognitive Neurology, Department of Neurosciences, Experimental Neurology, and Leuven Brain Institute (LBI), KU Leuven, Leuven 3000, Belgium; Alzheimer Research Centre KU Leuven, Leuven Brain Institute, KU Leuven, Leuven 3000, Belgium; Department of Electrical Engineering, ESAT/PSI, KU Leuven, Leuven 3000, Belgium; Medical Imaging Research Center, KU Leuven, Leuven 3000, Belgium; Department of Human Genetics, KU Leuven, Leuven 3000, Belgium; Department of Paediatrics, Murdoch Children’s Research Institute, Melbourne, Victoria 3052, Australia; Laboratory for Cognitive Neurology, Department of Neurosciences, Experimental Neurology, and Leuven Brain Institute (LBI), KU Leuven, Leuven 3000, Belgium; Alzheimer Research Centre KU Leuven, Leuven Brain Institute, KU Leuven, Leuven 3000, Belgium; Neurology Department, University Hospitals Leuven, Leuven 3000, Belgium

**Keywords:** genetic frontotemporal dementia, structural MRI, tensor-based morphometry, brain segmentation, size, shape

## Abstract

Traditional methods for detecting asymptomatic brain changes in neurodegenerative diseases such as Alzheimer’s disease or frontotemporal degeneration typically evaluate changes in volume at a predefined level of granularity, e.g. voxel-wise or in a priori defined cortical volumes of interest. Here, we apply a method based on hierarchical spectral clustering, a graph-based partitioning technique. Our method uses multiple levels of segmentation for detecting changes in a data-driven, unbiased, comprehensive manner within a standard statistical framework. Furthermore, spectral clustering allows for detection of changes in shape along with changes in size. We performed tensor-based morphometry to detect changes in the Genetic Frontotemporal dementia Initiative asymptomatic and symptomatic frontotemporal degeneration mutation carriers using hierarchical spectral clustering and compared the outcome to that obtained with a more conventional voxel-wise tensor- and voxel-based morphometric analysis. In the symptomatic groups, the hierarchical spectral clustering-based method yielded results that were largely in line with those obtained with the voxel-wise approach. In asymptomatic *C9orf72* expansion carriers, spectral clustering detected changes in size in medial temporal cortex that voxel-wise methods could only detect in the symptomatic phase. Furthermore, in the asymptomatic and the symptomatic phases, the spectral clustering approach detected changes in shape in the premotor cortex in *C9orf72*. In summary, the present study shows the merit of hierarchical spectral clustering for data-driven segmentation and detection of structural changes in the symptomatic and asymptomatic stages of monogenic frontotemporal degeneration.

## Teaser

Bruffaerts, Gors *et al*. studied structural brain changes at the asymptomatic stage of monogenic frontotemporal degeneration. They used hierarchical spectral clustering for MRI segmentation to detect changes at different levels of granularity. In asymptomatic *c9orf72* expansion carriers additional structural brain changes were observed in comparison to conventional methods.

## Graphical Abstract

**Graphical abstract:**
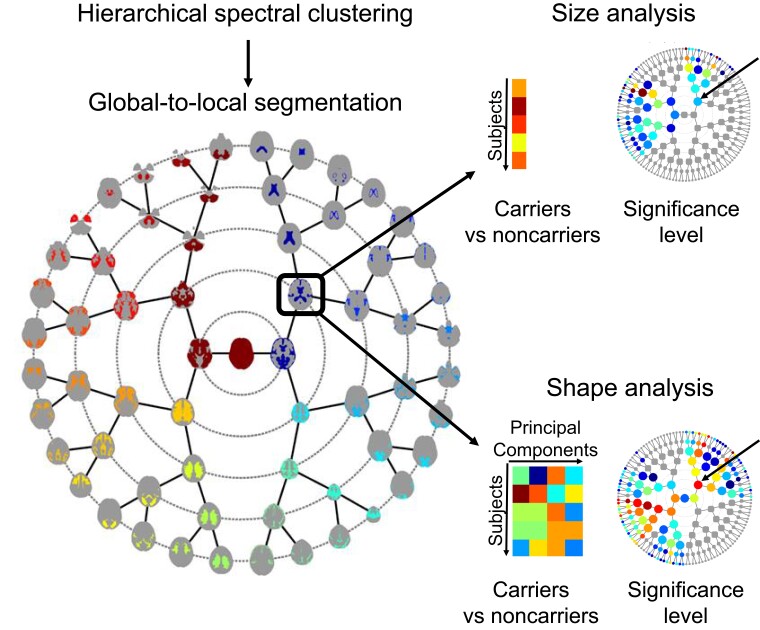

## Introduction

In autosomal-dominant neurodegenerative disease, anatomical changes in brain structure occur years before symptom onset.^1–4^ In frontotemporal degeneration (FTD), the most common autosomal mutations are chromosome 9 open reading frame 72 (*C9orf72*), progranulin (*GRN*), and microtubule-associated protein tau (*MAPT*). Large multicentre prospective cohorts such as the Genetic Frontotemporal dementia Initiative (GENFI) and the ARTFL-LEFFTDS Longitudinal Frontotemporal Lobar Degeneration study have led to the discovery of asymptomatic brain changes in autosomal-dominant FTD.^3^ This is important for the development of presymptomatic therapies to determine the optimal timing of a study intervention and to evaluate study drug effects.

In previous studies, structural changes were determined with either voxel-wise comparisons^4–6^ or an atlas-based approach using regions of interest.^3,7,8^ In an atlas-based approach, the extent of the volumes of interest is defined a priori, e.g. at the lobar level or at the level of individual regions of the atlas within a lobe. A landmark paper reported grey matter volumetric changes in the asymptomatic stage in monogenic FTD^3^ and related the volume-based morphometric changes to the expected time of symptom onset. In that study, the segmentation was automated and based on atlas propagation and fusion.^9^ The volumes consisted of frontal, temporal, parietal, and occipital lobe, insula, as well as thalamus, striatum, hippocampus, and amygdala. When all mutation carriers were grouped, 10 years before the expected symptom onset, the insula and the temporal lobe started to show volume decreases, and 5 years before symptom onset, the frontal and parietal lobe and all subcortical structures. The first changes were seen in *C9orf72* expansion carriers in the thalamus, insula and posterior cortical regions 25 years before the predicted time of onset.

Subsequent volumetric studies using a different methodology yielded findings that were largely in line with the Rohrer *et al*.^3^ report. Two of these studies^5,6^ used voxel-based morphometry (VBM) with statistical parametric mapping (SPM). Cash *et al*.^6^ reported a voxel-wise comparison between asymptomatic and symptomatic carriers respectively, and non-carriers. In asymptomatic *C9orf72* expansion carriers, atrophy was documented in the thalamus, right superior posterior cerebellum, superior temporal and inferior frontal regions. Changes were subtle in the other asymptomatic carriers: in *GRN* and *MAPT* carriers, no areas survived correction for multiple comparisons. In a small group of asymptomatic *C9orf72* carriers, Lee *et al*.^5^ reported volume loss in the insula, anterior cingulate, striatum and the medial pulvinar of thalamus compared to controls. A fourth study was based on a region-based analysis of cortical thickness in *C9orf72* carriers versus non-carriers from the same family.^10^ Thinning of the primary motor cortex was observed in patients with ALS but not in asymptomatic carriers. Cury *et al*.^8^ studied multivariate shape analysis using a different method, large diffeomorphic deformation metric mapping. Longitudinal changes were studied in an a priori defined region, namely the thalamus. Shape changes in the anterior portion of the thalamus were detected in asymptomatic mutation carriers. Finally, a recent study increased the resolution of subcortical anatomy using atlas propagation and fusion.^7^ Here, in the *C9orf72* expansion carriers the earliest volume changes were in thalamic subnuclei (pulvinar and lateral geniculate), cerebellum and hippocampus (particularly presubiculum and CA1), amygdala and hypothalamus.

In the present study, we implemented a recently proposed brain segmentation method based on hierarchical spectral clustering. Spectral clustering^11^ is a graph-partitioning technique separating vertices into segments. We have applied it before to study complex morphological changes, i.e. changes in brain shape and facial morphology as a function of complex genetic traits^12,13^ and to study structural MRI changes in Alzheimer’s disease.^14^ Hierarchical spectral clustering refers to spectral clustering at successive levels of granularity, from a global to a local level of segmentation. It may offer a number of advantages for the application at hand. A priori the level of segmentation that optimally captures the structural differences between carriers and non-carriers is unknown. Hierarchical spectral clustering allows one to segment the brain in a data-driven manner at different levels of granularity. In the present study, this segmentation is performed in a binary manner from level to level for a total of eight levels. The global-to-local segmentation can be likened to a variation of the spatial zoom, however, with the important characteristic that the spatial zooming at each level is determined exclusively by the similarity of features within a segment. The first levels correspond to a global view and are sensitive to more distributed general changes. The highest levels correspond to a multi-focal view optimally suited for more local changes. It is a priori unclear which degree of granularity would be optimal to detect the first changes. It may be that subtle changes are distributed and best detected when analyzing larger structures, e.g. whole brain atrophy with widening of the CSF or entire lobes as in Rohrer *et al*.^3^ Alternatively, more specific anatomical regions such as medial pulvinar^6^ may be affected first in a selective manner. With hierarchical spectral clustering the brain is partitioned from a global-to-local spatial scale, without the need to define the scale a priori. This is then coupled with a comprehensive statistical approach that takes into account the amount and interdependence of the comparisons at the multiple levels, i.e. in the current report false discovery rate (FDR). Second, our approach incorporated an analysis of different aspects of the segmental structural pattern, namely size and shape. Previous studies focused exclusively on the identification of between-group differences in segmental size. However, size alone ignores local patterns of co-variation that do not change the total size in a segment.

We evaluated the merits of this processing pipeline in GENFI and investigated whether the recent method could provide additional information about structural changes in (a)symptomatic carriers of FTD mutations at different levels of granularity. We determined the significant differences between the asymptomatic and symptomatic carriers, respectively, versus the non-carriers at the level of each segment from the global-to-local segmentation.

## Material and methods

### Participants

Data used in the preparation of this article were obtained from the GENFI (https://www.GENFI.org/). This large multicentre cohort focuses specifically on members of families where a known FTD gene mutation occurs, mainly *C9orf72* expansions (presence of >30 repeats), *MAPT* mutations and *GRN* mutations. GENFI recruits controls and asymptomatic carriers based on their genetic relation to the FTD patients, as well as FTD patients from (currently) 24 centres. By recruiting first-degree relatives, population stratification can be controlled for. The study was locally approved by the UZ/KU Leuven Ethics Committee for Research. After receiving a complete description of the study protocol, all participants or their legal representatives provided written informed consent in accordance with the Declaration of Helsinki.

At the time of the third data freeze, a total of 690 participants had been recruited across all centres. Scans from 55 participants were excluded because of missing data (50) or imaging artefacts upon visual inspection (5). Volumetric T_1_-weighted MRI scans of 118 symptomatic carriers, 267 asymptomatic carriers and 250 non-carriers were included for the present study. The baseline demographics of the 635 participants are given in Table 1, and the clinical information of the patients is reported in Table 2. Two-way ANOVA’s with mutation and genetic status (non-carriers, asymptomatic, symptomatic) were performed to asses between-group differences in age, sex and education level. For age, main effects of mutation (F(2,630) = 7.21, *P* < 0.001) and genetic status (F(2,630) = 95.1, *P* < 0.001) were observed, without interaction (P_interaction_: 0.67). *Post hoc* testing revealed that, as expected, the symptomatic carriers were older than the non-carriers and asymptomatic carriers (Tukey–Kramer *P* < 0.05). *MAPT* participants were younger than other mutation groups. There was also a significant main effect of status when modelling education (F(2,630) = 15.52, *P* < 0.001), with *post hoc* testing showing that the symptomatic carriers on average had fewer years of education than the asymptomatic carriers and non-carriers. There was also a main effect of status on sex in our cohort (F(2,630) = 5.62, *P* = 0.004), with more male participants in the symptomatic carrier group compared with the asymptomatic carriers and non-carriers groups. In all our analyses, age, sex, family membership and study site were included as covariates of no interest (analogous to Cash *et al*.^6^).

**Table 1:** Baseline demographics of the participants of the GENFI cohort used in this study

	**Non-carriers**	**Asymptomatic carriers**	**Symptomatic carriers**
***C9orf72***			
N (M:F)	85 (37: 48)	90 (56: 34)	56 (35: 21)^a^
Age	46.9 (13.8) (23–76)	45 (12.4) (20–68)	64.9 (7.6) (47–78)^a^
Education	13.7 (3.1) (5–20)	14.1 (2.9) (5–20)	12.6 (4) (5–22)^a^
***GRN***			
N (M:F)	125 (52: 73)	129 (45: 84)	42 (20: 22)^a^
Age	47.9 (14.3) (19–86)	46.6 (11.7) (20–76)	63.3 (9.1) (33–79)^a^
Education	14.1 (3.9) (5–24)	14.5 (3.5) (8–24)	10.8 (3.9) (5–18)^a^
***MAPT***			
N (M:F)	40 (21: 19)	48 (19: 29)	20 (13: 7)^a^
Age	44.3 (12.6) (20–71)^a^	40.8 (10.6) (21–74)^a^	57.3 (7.7) (38–69)^a^
Education	13.9 (3.4) (5–24)	14 (3.2) (5–20)	13.1 (4.2) (5–20)^a^

number of subjects, *N* (M:F); age and education (years), mean (std) (min–max); *N,* number; M, males, F, females.

^a^ significant difference from other groups with Tukey–Kramer *P* < 0.05.

**Table 2:** Clinical information of the patients of the GENFI cohort in this study

	*C9orf72* (*n* = 56)	*GRN* (*n* = 42)	*MAPT* (*n* = 20)
**Clinical diagnosis**			
bvFTD	73.21% (41)	52.38% (22)	100% (20)
FTD-ALS	8.93% (5)	—	—
ALS	7.14% (4)	—	—
PPA	5.36% (3)	40.48% (17)	—
CBS	—	4.76% (2)	—
PSP	1.79% (1)	—	—
Dementia-NOS	3.57% (2)	2.38% (1)	—

**Age at onset**	59.3 (8.7) (40–74)	61.2 (7.8) (48–77)	52.3 (6.2) (37–66)
**Disease duration**	5.4 (4.2) (0.8–20.4)	2.8 (1.9) (0.1–10.1)	5.0 (4.8) (0.2–17.4)
**MMSE (/30)**	23.3 (6.2) (0–30)	20.0 (6.1) (7–29)	25.6 (4.1) (16–30)
**CBI-R (/180)**	65.7 (30.3) (5–129)	60.3 (32.3) (11–126)	59.0 (37.6) (4–120)
**FRS (0-100%)**	36.8% (26.6%) (0–97%)	40.5% (26.3%) (4–97%)	49.2% (30.1%) (7–100%)
**ALSFRS-R (/48)**	37.5 (8.5) (20–45)	N/A^a^	N/A^a^

Clinical diagnosis: phenotype: percentage within genetic group (N); mean age at onset and disease duration in years (std) (min–max); MMSE, Mini-Mental State Examination; CBI-R, Cambridge Behavioural Inventory—Revised, FRS, FTD Rating Scale; ALSFRS-R, ALS Functional Rating Scale-Revised, for MMSE, CBI-R, FRS and ALSFRS-R the mean score is reported and (std) (min–max). bvFTD, behavioural variant of frontotemporal degeneration; ALS, amyotrophic lateral sclerosis; PPA, primary progressive aphasia; NOS, not otherwise specified; PSP, progressive supranuclear palsy; CBS, corticobasal syndrome.

^a^ only administered if a patient is diagnosed with (FTD-)ALS.

### Acquisition and preprocessing of the MRI data

#### Volumetric MRI acquisition

The GENFI imaging protocol comprises a set of standardized MRI sequences to be used across the different study sites. In the majority of participants baseline volumetric T1-weighted MRI were acquired using 3 T scanners (*n* = 587) [GE (25), Philips (242), Siemens (320)]. In the remainder, when there was no 3 T scanner available at the centre, scans were acquired on 1.5 T (*n* = 48) [GE (9), Siemens (39)] scanners.

#### Preprocessing

Tensor-based morphometry (TBM) maps were generated using the CAT12 toolbox (Structural brain mapping group, Jena, Germany, http://www.neuro.uni-jena.de/cat), an extension of SPM12 (Wellcome Trust Centre for Neuroimaging, London, UK, http://www.fil.ion.ucl.ac.uk/spm). Segmentation was performed in CAT12 using a default tissue probability map. Local adaptive segmentation was used at default strength (medium) and Diffeomorphic Anatomical Registration Through Exponentiated Lie Algebra^15^ was used for registration to the default template (IXI555 MNI152). The determinant of the gradients of the deformation field in every voxel resulted in the Jacobian maps, encoding the structural information that was used for tensor-based image segmentation. Voxel size was set at 1.5 mm (isotropic) after internal resampling at 1 mm. Images were smoothed using a 6 × 6 × 6 mm^3^ Gaussian kernel (for comparison to Cash *et al*.^6^). Quality of the raw data was verified by means of the CAT12 Image Quality Ratio (IQR) combining measures of noise, inhomogeneity and resolution.^16^ Quality of the segmentation step was verified by estimating sample homogeneity of the Jacobian maps using the CAT12 toolbox, which was calculated by correlating each individual map to the rest of the sample.

### Segmental tensor-based morphometry

#### Global-to-local segmentation

Global-to-local tensor-based segmentation was performed using hierarchical spectral clustering^12–14^ based on the Jacobian maps derived from the scans of all participants (symptomatic and asymptomatic carriers, as well as non-carriers, Supplementary Figure 1). We included the scans of all participants, whether they were carriers or not. The segmentation of brain shape (estimated from Jacobian maps) in a population sample should be stable and reproducible. Since the proposed segmentation was defined at the level of a population (and not at the level of an individual image), the population sample size becomes crucial and therefore the segmentation was defined on the complete data available within the GENFI cohort. Importantly, in our case no categorical disease labels were used as the segmentation was unsupervised.

Spectral clustering aims to organize voxels into clusters with high within-cluster and low between-cluster data relations. Specifically, this was done using the (spectral) eigen-decomposition of a square affinity matrix A (number voxels × number voxels) expressing all pair-wise data relations.^11^ In this work (and following Gors *et al*.^14^), based on all 635 MRI scans, the pair-wise relationship between two voxels *i* and *j* was set equal to: A(*i,j*) = 0.5(corr(J*_i_*, J*_j_*) + 1), with J*_i_* the Jacobian vector over each subject’s Jacobian map at the *i*^th^ voxel. Negative/positive correlations generated a low/high affinity between voxel pairs. Pairs with high affinity are likely to cluster together, and pairs with low affinity are likely to cluster differently. Subsequently, the matrix A was transformed into a Laplacian matrix *L*=*D*^-1/2^*AD*^-1/2^, from which the eigenvectors associated with the *k* (*k* = 2, for a split into two clusters) highest eigenvalues were taken. D is a diagonal matrix, where d*_i, i_* represents the sum of the *i*^th^ column of the matrix A. This transformation of A into L followed by an eigen-decomposition enhances the (dis)similarities of voxel pairs in A, and therefore simplifies the partitioning problem of voxels into clusters. In the eigenvector space, the Euclidean distance, serves as a proxy for the affinity, and drives a k-means clustering to partition the vertices into two clusters. Computationally, these operations are not straightforward since the construction of the matrices A and L was not possible due to memory constraints when working with over 5 × 10^5^ voxels. This was solved using Nyström^17^ which is a technique that simultaneously estimates the Laplacian and the eigenvectors of a large-scaled matrix using only a random subset of its columns. Finally, due to the random behaviour in k-means and Nyström, a two step-robustness procedure was implemented. The first step averaged multiple Nyström estimations (*n* = 50) resulting from randomly different voxel subsets. In the second step, *k*-means was repeated 50 times and the produced clusters are merged into a single partitioning with a normalized vote-weighting scheme.^18^

**Figure 1:**
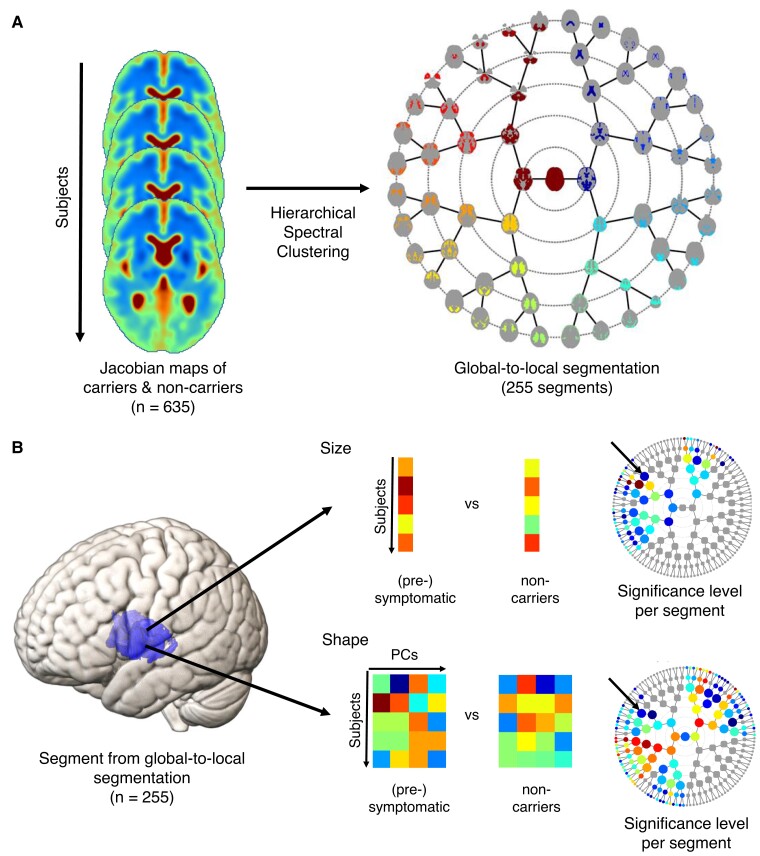
**Schematic of workflow.** (**A**) Global-to-local segmentation with visualization of the 1*st* to 6*th* level of the hierarchical segmentation with circular dendrograms (3*th* to 8*th* level is visualized in Supplementary Figure 2 for completeness and 3D images were uploaded as Supplementary data). Intra-segment voxels are randomly coloured according to their position in hierarchical diagrams. For each segment, the transversal plane with the highest number of intra-segment voxels is visualized. (**B**) Statistical analysis of size and shape components per segment, calculated respectively by means of a Student’s *t* test comparing the average Jacobians and CCA comparing the principal components (PCs), and link to the circular dendrogram summarizing significance levels across segments (−log_10_*P*-value).

A hierarchical spectral clustering was obtained by sequentially splitting a segment into two disjoint segments, whereby each new segment independently of the other is further partitioned into two and so on. From one level to the next, a binary segmentation was performed, i.e. two segments in the lower level for every parent segment, for a total of eight levels. The first level contained the entire intracranial volume. The second level consisted of two segments, going up to 2^7^ segments at the highest eighth level (Fig. 1A).

#### Statistical analyses

Jacobians within each segment of the global-to-local segmentation were summarized as segmental size features and segmental shape coefficients. Segmental size was computed as the average Jacobian across all voxels within a segment. Segmental shape coefficients were calculated as follows: first, we corrected for segmental size by dividing the Jacobian values of a segment by its size (i.e. the average Jacobian). After this division, the shape space was computed using principal component analysis (PCA, singular value decomposition). Besides the resulting dimensionality reduction, PCA has the advantage that linear combinations of the principal components enable a reconstruction of the original space. Parallel analysis^19^ was used to determine the significant principal components.

Within each segment structural differences between the asymptomatic FTD mutation carriers versus non-carriers, and symptomatic FTD mutation carriers versus non-carriers were determined to study the potential of the new method (Fig. 1B). Hierarchical spectral clustering is then coupled with a comprehensive statistical approach that takes into account the amount and interdependence of the comparisons. Between-group differences in size were analyzed using Student’s *t* tests (assuming equal variance). Differences in shape, which is a multivariate feature, were tested using canonical correlation analysis (CCA). Testing was performed separately on all 255 segments (of the eight levels) of the hierarchical segmentation. The FDR-adjusted significance threshold was computed at *P* = 0.0007 (‘dep’,^20^ (*α* < 0.05)). The threshold considers the number of contrasts (*n* = 6), the number of form features (size and shape, *n* = 2), as well as the number of segments (*n* = 255). It means that 12 tests on 255 segments are performed simultaneously corresponding to the abovementioned P threshold. Age, sex, image acquisition site and family membership were added to all analyses as covariates of no interest.^6^ Local deformations were estimated using the Jacobian determinant, whereas ignoring the affine part of the deformation field. Thus, additional correction for total intracranial volume was not required because only non-linear deformation was taken into account.^21^

### Univariate morphometric analysis

The global-to-local segment results were compared with those obtained for the same contrasts using well-established (univariate) VBM and TBM to compare the sensitivity of our method to the standard procedure. Using SPM12, symptomatic carriers and asymptomatic carriers of every genetic group were compared with non-carriers using a one-way between-subject ANOVA. The same covariates (age, sex, acquisition site and family membership) were used as in the multivariate approach. Total intracranial volume was added as a covariate for VBM. The FDR-adjusted significance threshold was computed at *P* = 0.0003 (‘dep’,^20^ (*α* < 0.05)), considering the number of contrasts (*n* = 6), the number of form features (size and shape, *n* = 2), as well as the number of segments (*n* = 255). For comparison to the hierarchical spectral clustering analysis, we only report clusters larger than 500 voxels because the smallest segment in the eighth level of segmentation contained 563 voxels.

### Exploratory analysis of global-to-local segment results

The patients presented with different clinical phenotypes depending on their mutation (Table 2). To determine whether all clinical phenotypes contributed equally to the global-to-local segment results in the symptomatic groups, we calculated thresholded maps for size and shape. The thresholded maps were calculated as follows: a binary voxel-wise map was generated for every level indicating which segments passed the preset FDR-corrected threshold. Maps were summed across all 8 levels, resulting a pattern for size and a pattern for shape for every mutation. The thresholded maps were used to weight the Jacobian values per voxel resulting in an average weighted Jacobian value for each individual. Within the symptomatic carriers, ANOVA’s were conducted for the *C9orf72* and *GRN* group to determine the effect of clinical phenotype (in the *MAPT* group, all symptomatic individuals exhibited the same phenotype, bvFTD).

Finally, we determined the relationship between the size and shape changes. The pair-wise Dice coefficient was calculated between every mutation group per genetic status (asymptomatic, symptomatic) to compare the overlap of the thresholded maps for size and shape. For the purpose of this comparison, the voxel-wise maps generated for every level were concatenated instead of summed to serve as binary input for calculation of the dice coefficients.

### Data and code availability

The input data can be requested as the third data freeze from the GENFI (http://www.GENFI.org/). SPM12 (https://www.fil.ion.ucl.ac.uk/spm/software/spm12/) and the CAT12 toolbox (http://www.neuro.uni-jena.de/cat/) are freely available online. The in- house Matlab routine to perform hierarchical spectral clustering can be downloaded from https://gitlab.kuleuven.be/u0064036/hierarchical-spectral-clustering.

## Results

### Data quality

Mean image quality was assessed as ‘good’ using the CAT12 IQR^16^ (mean IQR: 2.15, s.d. 0.31). A two-way ANOVA with mutation and genetic status (non-carriers, asymptomatic, symptomatic) to assess between-group differences in IQR showed differences for genetic status (F(2,630) = 29.6, *P* < 0.001). *Post hoc* testing revealed that the symptomatic individuals had lower image quality, but this effect disappeared when age was added as a covariate (genetic status: *P* = 0.213, age: *P* = 0.005).

Sample homogeneity for the Jacobian maps was on average 0.85 (s.d. 0.02). A two-way ANOVA with mutation and genetic status to assess between-group differences in homogeneity showed differences for mutation group (F(2,630) = 7.96, *P* < 0.001). *Post hoc* testing demonstrated that the *MAPT* group had higher sample homogeneity, but this effect disappeared when age was added as a covariate (mutation: *P* = 0.710, age: *P* = 0.288) (recall that the *MAPT* group was younger).

### Global-to-local segmentation

The hierarchical spectral clustering algorithm resulted into 255 segments across the eight hierarchical levels. Fig. 1A displays the first six levels of the segmentation as orbits of a dendrogram: the level of granularity increases when moving towards the outer orbits. The first level corresponds to the entire intracranial compartment. The second level results from a binary segmentation into a compartment largely made up of the CSF and a brain compartment. The projection of the segments of the levels 3–8 onto the normalized brain is shown in Supplementary Figure 2.

### Hypothesis testing

**Figure 2:**
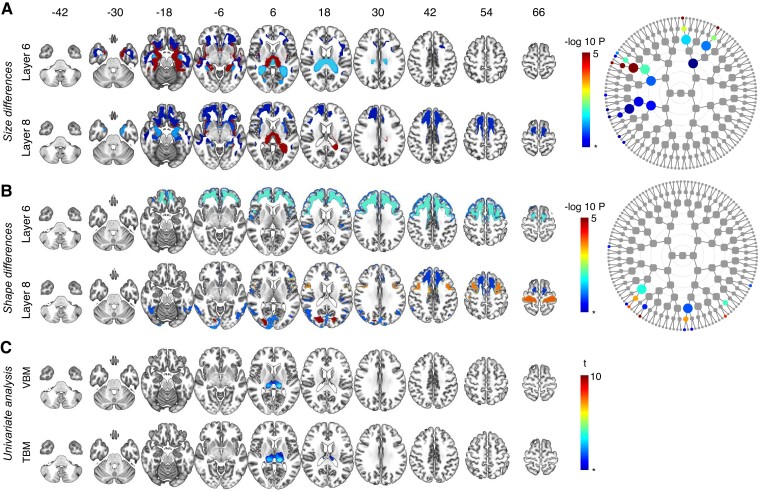
**Asymptomatic carriers *C9orf72* versus non-carriers.** Global-to-local segment results for **A** size and **B** shape and their respective dendrograms. Asterisk indicates FDR-adjusted significance (dep) *P* = 0.0007, −log *P* = 3.17, results below the FDR-adjusted significance threshold are not illustrated. Nodes can be linked to their spatial coverage via Fig. 1A. Results from other levels are shown in Supplementary Figure 3. (**C**) VBM and TBM analysis: asterisk indicates FDR-adjusted significance (dep) *P* = 0.0003, t = 3.46. Note that the by convention, only atrophy is visualized so widening of the ventricles cannot be assessed from the univariate results.

**Figure 3:**
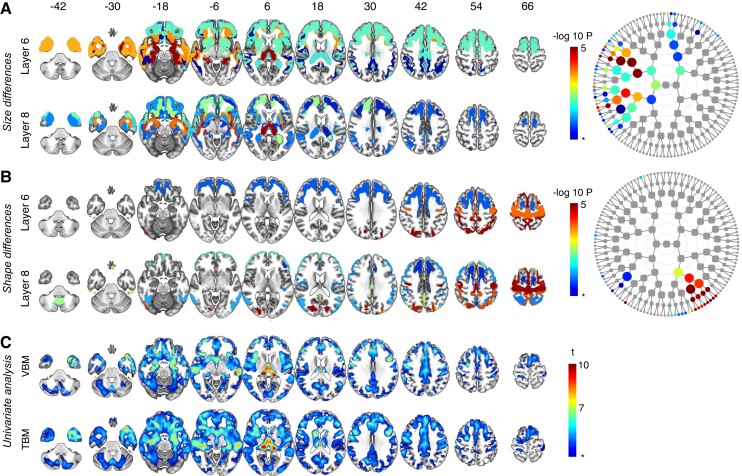
**Symptomatic carriers *C9orf72* versus non-carriers.** Global-to-local segment results for **A** size and **B** shape and their respective dendrograms. Asterisk indicates FDR-adjusted significance (dep) *P* = 0.0007, −log *P* = 3.17, results below the FDR-adjusted significance threshold are not illustrated. Nodes can be linked to their spatial coverage via Fig. 1A. Results from other levels are shown in Supplementary Figure 4. (**C**) VBM and TBM analysis: asterisk indicates FDR-adjusted significance (dep) *P* = 0.0003, *t* = 3.46.

**Figure 4:**
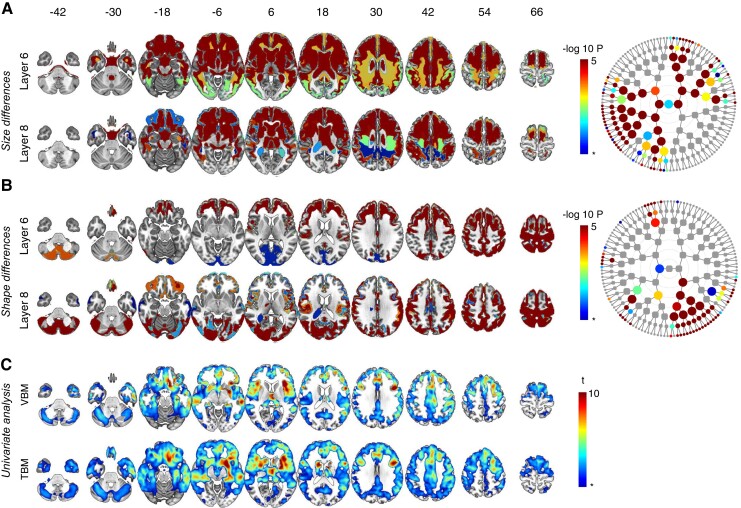
**Symptomatic carriers *GRN* versus non-carriers.** Global-to-local segment results for **A** size and **B** shape and their respective dendrograms. Asterisk indicates FDR-adjusted significance (dep) *P* = 0.0007, −log *P* = 3.17, results below the FDR-adjusted significance threshold are not illustrated. Nodes can be linked to their spatial coverage via Fig. 1A. Results from other levels are shown in Supplementary Figure 5. (**C**) VBM and TBM analysis: asterisk indicates FDR-adjusted significance (dep) *P* = 0.0003, *t* = 3.46.

**Figure 5:**
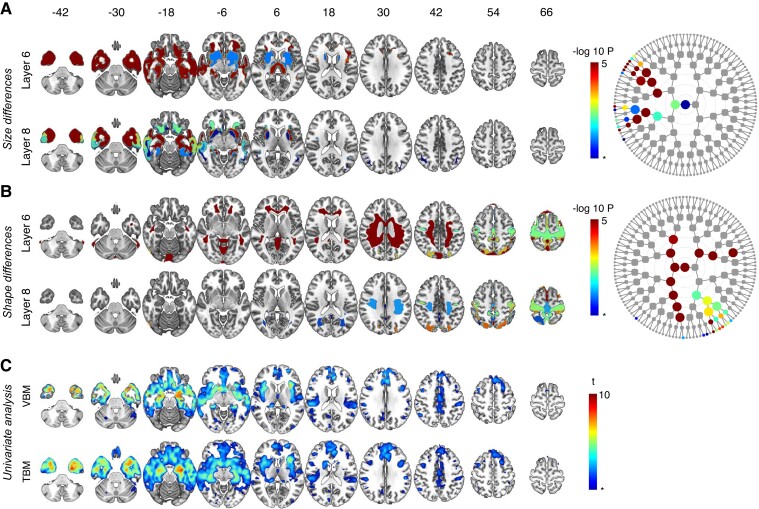
**Symptomatic carriers *MAPT* versus non-carriers.** Global-to-local segment results for **A** size and **B** shape and their respective dendrograms. Asterisk indicates FDR-adjusted significance (dep) *P* = 0.0007, −log *P* = 3.17, results below the FDR-adjusted significance threshold are not illustrated. Nodes can be linked to their spatial coverage via Fig. 1A. Results from other levels are shown in Supplementary Figure 6. (**C**) VBM and TBM analysis: asterisk indicates FDR-adjusted significance (dep) *P* = 0.0003, *t* = 3.46.

For each of the 255 segments, the statistical significance of a segmental size and shape difference between the asymptomatic or the symptomatic carriers of the FTD mutations respectively versus non-carriers was determined. The results are projected on the dendrograms of Figs. 2–5. These overlays represent the same segmentation as shown in Fig. 1A but now the colours at each position in the dendrogram depict the segmental *P-*value, when statistically significant. Only results surviving FDR correction (‘dep’^20^) are reported. These representations allow us to examine if the genetic mutation has a relatively global or relatively local impact on brain structure depending on the position of the effect on the dendrogram. Because of the hierarchical organization of the segmentation, the results found in parent and child segments are derived on either the ensemble (parents) or a partition (children) of the same data. The dendrograms on the right-hand side aid in identifying the level of optimal granularity, i.e. the level at which the result is the strongest. Evidently, significant results may sometimes occur in parent or child segments of the segment with optimal granularity, caused by these shared datapoints.

#### C9orf72 asymptomatic carriers versus non-carriers

Using spectral clustering, significant size differences were found in the posteromedial thalamus but also in the medial temporal cortex, the striatum, the insula and orbitofrontal cortex (Fig. 2A). In asymptomatic carriers of *C9orf72* expansions versus non-carriers, shape changes were observed in the motor cortices, inferior frontal gyri and in the white matter of the occipital lobes (Fig. 2B). A univariate voxel-wise VBM and TBM analysis showed posterior thalamic atrophy but no other abnormalities in *C9orf72* asymptomatic carriers (Fig. 2C).

#### C9orf72 symptomatic carriers versus non-carriers

The global-to-local segment results were topographically similar to those found in the asymptomatic group, but more pronounced size changes were observed when comparing the symptomatic carriers to the non-carriers (Fig. 3A). Similarly, shape changes in the motor cortex were highly significant in the group of symptomatic carriers (Fig. 3B), accompanied by widespread changes predominantly in the frontal lobes (compared with non-carriers) but extending to other regions as well. A univariate voxel-wise VBM and TBM analysis demonstrated extensive atrophy in the frontal and anterior temporal lobes, as well as the basal ganglia compared with the non-carriers (Fig. 3C).

#### GRN carriers versus non-carriers

In the asymptomatic *GRN* carriers, univariate analysis and global-to-local segment results did not reveal any differences with the non-carriers. Using spectral clustering, widespread size differences were found at all segmental levels when comparing symptomatic carriers to non-carriers, with a predominance in the prefrontal grey and white matter (Fig. 4A). The spectral clustering analysis showed that symptomatic *GRN* mutation carriers displayed widespread shape changes in the frontal and parietal regions (including the precuneus), as well as the cerebellum (Fig. 4B). The changes in symptomatic carriers versus non-carriers were already detected at the 2*nd* global level of segmentation and persisted at higher levels. By means of univariate analysis, *GRN* symptomatic carriers demonstrated extensive atrophy in the frontal and anterior temporal lobes, as well as the basal ganglia compared with non-carriers (Fig. 4C).

#### Microtubule-associated protein tau carriers versus non-carriers

Univariate analysis and spectral clustering did not reveal any difference in the asymptomatic *MAPT* carriers versus the non-carriers. The size analysis in the symptomatic carriers revealed changes at all segmental levels compared with non-carriers, with prominent atrophy in the anterior temporal lobe, frontobasal region and basal ganglia (Fig. 5A). Symptomatic carriers displayed shape changes in the motor cortices, as well as in the white matter compared with non-carriers (Fig. 5B). Changes were identified across all levels of segmentation and focused on the white matter. The VBM and TBM atrophy pattern in *MAPT* symptomatic carriers compared with non-carriers was slightly different compared with the *C9orf72* and *GRN* groups with the most pronounced atrophy in the anterior temporal lobes, frontobasal region and basal ganglia (Fig. 5C).

### Effect of clinical phenotype and comparison across size and shape results

**Figure 6:**
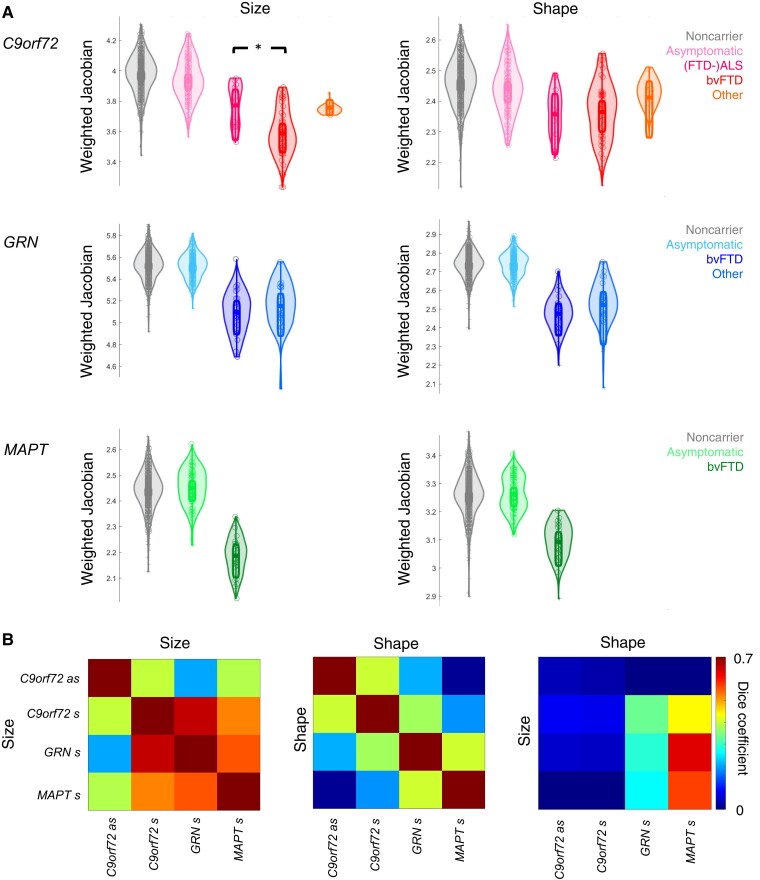
**Stratification per disease phenotype and size versus shape results.** (**A**) Jacobians weighted by means of the thresholded maps for size and shape changes per genetic group. (*) indicates between-phenotype differences within the symptomatic groups (*Post hoc* comparison Tukey–Kramer *P* < 0.05), note that we did not include the non-carriers and asymptomatic carriers in this statistical comparison. Plots were generated using the Robust Statistic Toolbox.^22^ (**B**) Pair-wise Dice coefficients between the thresholded maps for (a)symptomatic *C9orf72* carriers (respectively a, s) and symptomatic *GRN* and *MAPT* carriers (s). Note that for size × size and shape × shape comparisons, the Dice coefficient on the diagonal is one (identical) but that the comparison between size and shape results in an asymmetrical matrix with values of <1 on the diagonal.

Within the thresholded maps for symptomatic participants, we tested whether the distinct clinical phenotypes differentially contributed to the size and shape differences (Fig. 6A). For the symptomatic *c9orf72* carriers, where size changes were found in the thalamus and frontotemporal cortex, differences were observed between the clinical phenotypes (F(2,53) = 6.58, *P* = 0.003). *Post hoc* testing revealed that the size changes were more pronounced in the bvFTD phenotype compared to the (FTD-)ALS phenotype (Tukey–Kramer *P* < 0.05). For shape changes, no differences were found between the different *C9orf72* phenotypes (F(2,53) = 0.66, *P* = 0.521). No differences between phenotypes were found for size or shape changes in the *GRN* group (*P* > 0.242).

Dice coefficients were calculated to compare the overlap between global-to-local segment results in the asymptomatic and symptomatic *C9orf72* carriers, and the symptomatic *GRN* and *MAPT* carriers (Fig. 6B). The overlap of the thresholded maps of asymptomatic *C9orf72* carriers was consistently highest with the symptomatic *C9orf72* carriers. Between all symptomatic groups, substantial overlap was found between the thresholded maps for size differences. We also calculated the overlap between thresholded maps of size and thresholded maps of shape differences: on average, their overlap was low (Fig. 6B, mean dice coefficient: 0.18).

## Discussion

We applied a recent method based on hierarchical spectral clustering to analyze a large multicentric data set of structural MRI scans obtained in symptomatic and asymptomatic autosomal-dominant FTD mutation carriers and non-carriers. In particular, in asymptomatic *C9orf72* expansion carriers, spectral clustering revealed changes in segmental size that more conventional methods detected only at the symptomatic stage. Furthermore, the method allows for analysis of shape and this demonstrated significant changes in regions known to be involved in *C9orf72* pathophysiology but not seen with the conventional methods. Third, spectral clustering revealed a large number of significant changes in the symptomatic phase of FTD mutations that closely corresponded to those seen also with the univariate approaches, thus suggesting that spectral clustering is sensitive to pick up these known effects as well as additional changes.

### Size and shape changes in asymptomatic individuals

In the asymptomatic phase of *C9orf72*, the novel method had a higher sensitivity than voxel-wise methods. These conventional methods only revealed abnormalities in the posteromedial thalamus (or medial pulvinar), in line with a number of previous reports,^5,6,23^ while our method additionally showed highly significant size changes in medial temporal, orbitofrontal and medial frontal cortex and insula. Atrophy in the pulvinar, which is part of the limbic system, has been linked to attentional deficits^5^ as well as altered pain perception^24^ in *C9orf72* carriers. Disrupted connectivity of the posteromedial thalamus has been correlated with more severe behavioural symptoms.^25^ Atrophy of the orbitofrontal cortex and insula was previously associated with impaired social cognition in *C9orf72* carriers.^26^ Atrophy of the medial temporal lobe has been observed in *C9orf72* carriers with the ALS-FTD phenotype, whereas it was absent in carriers presenting with ALS.^27^ Here, the size differences were most prominent in symptomatic *C9orf72* carriers presenting with bvFTD compared with other phenotypes. This is consistent with early size changes occurring in regions related to the bvFTD phenotype in asymptomatic individuals.

The location of changes detected in asymptomatic *C9orf72* expansion group corresponded to the location of most changes found with conventional TBM/VBM in the symptomatic group and in the literature.^3,6,26^ This colocalization of changes in the asymptomatic group with changes in the symptomatic group increases the likelihood that the method identifies the earliest FTD-related changes, although longitudinal data is needed to confirm this hypothesis. Interestingly, highest significance in medial temporal cortex is reached at the sixth level of segmentation and decreases at the eight level. This may explain why it remains below the detection threshold of voxel-based analyses and demonstrates the value of varying levels of granularity in the analysis as some changes may be more readily detectable at a lower level of resolution. Our findings, which replicate results obtained using a recent detailed atlas-based method,^7^ may reflect the fact that the medial temporal cortex is affected relatively early. The identification of the spatial extent of hippocampal involvement exemplifies an important advantage of our method which examines brain changes at different levels of granularity based on a data-driven partitioning algorithm.

There was also a clear advantage of analyzing changes in shape, which resulted an atrophy pattern distinct from the size changes. In asymptomatic as well as symptomatic *C9orf72* carriers, changes in shape were detected in premotor cortex. There is a known vulnerability of motor cortex in this disease which may cause either FTD or ALS.^28^ Using fluorine 18-labeled fluorodeoxyglucose positron emission tomographic imaging, hypermetabolism was observed in this region in the majority of asymptomatic *C9orf72* expansion carriers compared to controls,^29^ possibly reflecting neuroinflammation. Given the location near the primary motor cortex and the vulnerability of the upper motor neuron system in *C9orf72*, there are strong neurobiological arguments to consider this difference in shape in the asymptomatic and the symptomatic *C9orf72* expansion carriers versus non-carriers as a true-positive effect. Furthermore, significant changes in shape were present in asymptomatic carriers in the lateral prefrontal cortex. These widespread prefrontal changes were more prominent at the sixth level of segmentation than at the eighth. An interesting next step would be to derive the segments from this analysis with the highest significance for detecting presymptomatic changes (which may be either the sixth or eighth level) and determine in an independent dataset how well asymptomatic individual cases can be discriminated from non-carriers based on the values obtained in these segments. More generally, a classifier could be trained on a training data set so that the best segments for classification are defined and then used in a test set.^30^ Such individual-level analyses could be used for early diagnosis, disease progression monitoring^31^ or to study the effect of new drugs.

In the asymptomatic *MAPT* and *GRN* mutation carriers no changes were found with the conventional or the novel methods. Cash *et al*.^6^ reported changes in these groups with VBM in the medial and anterior temporal lobe and the posterior insula, respectively, but these were limited in extent and were only present at a low significance threshold. Bocchetta *et al*.^7^ reported discrete abnormalities in these genetic groups at a lower significance threshold (FDR correction using ‘pdep’^32^), which were also present in our data set at this threshold: we observed size changes in the precuneus in *MAPT* asymptomatic carriers and shape changes in the striatum of *GRN* asymptomatic carriers. One can only speculate about the reasons why presymptomatic changes are much harder to detect in these two mutation groups than in *C9orf72* expansion carriers. In *MAPT,* the phase preceding the symptomatic phase may be relatively rapidly progressive^33^ so that asymptomatic carriers who are farther removed from symptom onset do not yet show detectable structural changes. In *MAPT*, grey matter atrophy was observed in a subset of asymptomatic carriers.^34^ Our statistical analyses, which test between-group differences, may not be sensitive to atrophy existing only in a small number of participants. In *GRN* the changes may be more asymmetrical and the method as it is currently implemented would not be able to detect these asymmetries.

### Size and shape changes in symptomatic individuals

In symptomatic *GRN* mutation carriers the pattern obtained with hierarchical spectral clustering matched that seen with the conventional methods. Furthermore, we found more extensive white matter size differences in the symptomatic *GRN* mutation carriers with spectral clustering than with conventional methods, most significantly so in frontal white matter. An increased volume of white matter hyperintensities has been reported before in symptomatic *GRN* mutation carriers mainly in the frontal and occipital lobe.^35,36^ In the asymptomatic *GRN* mutation carriers, a correlation has been found between white matter hyperintensity volumes and time to expected disease onset.^36^ These hyperintensities are associated with grey matter atrophy and biomarker changes (GFAP and NfL).^37^ In symptomatic *MAPT* mutation carriers, the well-known prominent involvement of the anterior temporal pole seen with conventional analyses^4,33^ was also observed with the hierarchical spectral clustering approach.

### Comparison to other segment-based methods

Image segmentation is important to help clinicians and researchers to focus on specific regions of the brain.^38^ In that sense, our hierarchical segmentation presents a global-to-local definition of regions to be analyzed statistically. This is a ‘divide and conquer’ strategy keeping the multiple testing burden into account in comparison to voxel-by-voxel-based analyses. Besides the hierarchical implementation the proposed segmentation is also different to related literature in two main aspects. First, related voxel-based clustering techniques operate at the level of an individual image, considering the voxels as a collection of datapoints to be grouped based on intensity.^38^ In contrast, the proposed clustering is defined at the level of a population, in which similarity between voxels is expressed by correlated variance or covariance of voxel features (shape coded by Jacobians) within the population sample. To the best of our knowledge, this is the first such population-based approach, for which some technical challenges related to data size and complexity were solved in Gors *et al*.^14^ Second, related voxel-based clustering techniques aim at segmenting anatomically or functionally defined regions, typically evaluated against manual expert segmentations. In contrast, the proposed segmentation does not aim to segment the brain into specifically known regions but instead the aim is to group into population correlated regions. The idea is to provide an ensemble of correlated datapoints within a single segment as input to statistical testing paradigms.^12,13^ Therefore, the segmentation proposed is not to be evaluated against expert segmentations but tested based on statistical power as illustrated in this work.

As expected, our results show a significant overlap with prior findings based on atlas-based methods.^3,7,8^ Furthermore, our approach shows how the use of different atlases containing regions of interests of different sizes, may lead to conflicting results. The fact that we offer a hierarchical overview of the significance levels in the form of the dendrogram, increases the insight into the spatial extent of structural changes irrespective of a predefined extent based on a particular atlas.

### Strengths and future directions

A benefit of a global-to-local segmentation is that it relaxes the strong dimensionality reduction burden at the global levels, which in isolation may lead to loss of information. Local volumetric patterns are representatively described (and analyzed) at the local levels. It is therefore not needed that the local variations are expressed in the form features of the global levels as well. Furthermore, complete form features at the global levels would introduce redundancy between the levels and actually counters the analyses of the global form effects that are equally of interest.

Further extensions and modifications to the hierarchical spectral clustering method can be foreseen. For instance, the choice of eight (fixed) levels was based on pragmatic reasons, allowing for sufficiently fine-grained analysis, on the one hand, and, on the other hand, avoiding an inflation of number of segments and therefore controlling the multiple testing burden. Importantly, this choice was made a priori. Conceivably, the number of levels can also be adapted to the features of the segments themselves, such as homogeneity. For some segments, such as CSF, the binary subdivision could stop already at a relatively early level when the segments exhibit homogeneity. Other segments, such as medial temporal cortex or thalamus, have such an intricate inherent structure that further segmentation beyond the eighth level when this is warranted by the homogeneity measure. A related matter is the binary nature of the division of the segments per level. Based on the segmental homogeneity one could easily imagine a division in multiple segments.

### Limitations

To date, we have demonstrated the merit of global-to-local segmentation in Alzheimer’s disease^14^ and here in FTD. The segments in which structural changes were linked to monogenic FTD, will require further out-of-sample validation in other genetic FTD cohorts before clinical translation is in order. In its current implementation, our technique (including segmentation, feature extraction and evaluated hypothesis) is not designed to detect asymmetry, which is typically present in the brains of the *GRN* carriers.^23^ Asymmetry detection asks for identification of group changes in intra-individual differences between the left and right hemispheres. A first step in making the approach adequate for this contrast is introducing a symmetric segmentation. The second step is defining size and shape features that measure the amount of asymmetry as differences between left and right. In the conventional shape space, the symmetric variations explain the major variability within the Jacobian maps, while the asymmetric variations have only minor contribution to it. Therefore, parallel analyses retain mainly the symmetrical-related PCA coefficients. An alternative is to subtract the Jacobian values of the left part of a segment from the corresponding right part of the segment before PCA training. The resulting shape coefficients represent the pattern of asymmetrical variability within the segment.

The current report evaluates the merits of the novel method at the group level. In clinical research, detection of change at the individual level is of increasing importance as it may inform selection of individuals for therapeutic trials. Hence, a relatively straightforward extension will be the application of a classifier based on segments derived based on hierarchical spectral clustering on single patient data. A consideration is that more study is needed on the relation between participant characteristics such as age (included as covariate here), education, disease severity, … and the structural changes observed in this report. Previous reports indicated that higher educational attainment relates to slower loss of grey matter over time in carriers of FTD mutations.^39^ Further investigation is not straightforward in the current data set because of multicollinearity between for instance age, education and disease severity (measured using CBI-R and FRS). It is possible that a single-case approach can not only rely on neuroimaging data but should also consider other individual characteristics.

Finally, even though the asymptomatic group of monogenetic FTD offers a unique insight into the early stages of the disease, the comparisons made here between the asymptomatic and symptomatic groups are cross-sectional and remain to be confirmed through longitudinal follow-up.

## Conclusions

To summarize, the merits of the hierarchical spectral clustering versus the conventional methods was most evident in the asymptomatic *C9orf72* carriers. This demonstrates the advantage of data-driven segments at varying degrees of granularity in their entirety rather than at a voxel level in this disease. In *MAPT* and *GRN* groups, global-to-local segment results converged with the focal changes also seen with the conventional univariate method.

## Supplementary Material

fcac182_Supplementary_Data

## Appendix I

### GENFI consortium authors

Sónia Afonso, Maria Rosario Almeida, Sarah Anderl-Straub, Christin Andersson, Anna Antonell, Silvana Archetti, Andrea Arighi, Mircea Balasa, Myriam Barandiaran, Nuria Bargalló, Robart Bartha, Benjamin Bender, Alberto Benussi, Sandra Black, Martina Bocchetta, Sergi Borrego-Ecija, Jose Bras, Marta Canada, Valentina Cantoni, Paola Caroppo, David Cash, Miguel Castelo-Branco, Rhian Convery, Thomas Cope, Giuseppe Di Fede, Alina Díez, Diana Duro, Chiara Fenoglio, Catarina B. Ferreira, Nick Fox, Morris Freedman, Giorgio Fumagalli, Alazne Gabilondo, Roberto Gasparotti, Serge Gauthier, Stefano Gazzina, Giorgio Giaccone, Ana Gorostidi, Caroline Greaves, Rita Guerreiro, Carolin Heller, Tobias Hoegen, Begoña Indakoetxea, Vesna Jelic, Lize Jiskoot, Hans-Otto Karnath, Ron Keren, Tobias Langheinrich, Maria João Leitão, Albert Lladó, Sandra Loosli, Carolina Maruta, Simon Mead, Lieke Meeter, Gabriel Miltenberger, Rick van Minkelen, Sara Mitchell, Katrina Moore, Jennifer Nicholas, Linn Öijerstedt, Jaume Olives, Sebastien Ourselin, Alessandro Padovani, Jessica Panman, Janne M. Papma, Georgia Peakman, Yolande Pijnenburg, Enrico Premi, Sara Prioni, Catharina Prix, Rosa Rademakers, Veronica Redaelli, Tim Rittman, Ekaterina Rogaeva, Pedro Rosa-Neto, Giacomina Rossi, Mar tin Rossor, Beatriz Santiago, Elio Scarpini, Sonja Schönecker, Elisa Semler, Rachelle Shafei, Christen Shoesmith, Miguel Tábuas-Pereira, Mikel Tainta, Ricardo Taipa, David Tang-Wai, David L Thomas, Paul Thompson, Hakan Thonberg, Carolyn Timberlake, Pietro Tiraboschi, Emily Todd, Michele Veldsman, Ana Verdelho, Jorge Villanua, Jason Warren, Carlo Wilke, Ione Woollacott, Elisabeth Wlasich, Henrik Zetterberg, Miren Zulaica

## Abbreviations

ALS =: amyotrophic lateral sclerosis
ALSFRS-R =: ALS functional rating scale-revised
CBI-R =: Cambridge Behavioural Inventory—Revised
*C9orf72* =: chromosome 9 open reading frame 72
CCA =: canonical correlation analysis
CBS =: corticobasal syndrome
FDR =: false discovery rate
FTD =: frontotemporal degeneration
GENFI =: Genetic Frontotemporal dementia Initiative
GFAP =: glial fibrillary acidic protein
*GRN* =: progranulin
*MAPT* =: microtubule-associated protein tau
MMSE =: Mini-Mental State Examination
Nfl =: neurofilament light chain
PPA =: primary progressive aphasia
PCA =: principal component analysis
PSP =: progressive supranuclear palsy
SPM =: statistical parametric mapping
TBM =: tensor-based morphometry
VBM =: voxel-based morphometry

## References

[1] Reiman EM , Quiroz YT, Fleisher AS, et al Brain imaging and fluid biomarker analysis in young adults at genetic risk for autosomal dominant Alzheimer’s disease in the presenilin 1 E280A kindred: A case-control study. Lancet Neurol. 2012;11(12):1048–1056. doi:10.1016/S1474-4422(12)70228-4 23137948 10.1016/S1474-4422(12)70228-4 PMC4181671

[2] Bateman RJ , Xiong C, Benzinger TLS, et al Clinical and biomarker changes in dominantly inherited Alzheimer’s disease. N Engl J Med. 2012;367(9):795–804. doi:10.1056/NEJMoa1202753 22784036 10.1056/NEJMoa1202753 PMC3474597

[3] Rohrer JD , Nicholas JM, Cash DM, et al Presymptomatic cognitive and neuroanatomical changes in genetic frontotemporal dementia in the genetic frontotemporal dementia initiative (GENFI) study: A cross-sectional analysis. Lancet Neurol. 2015;14(3):253–262. doi:10.1016/S1474-4422(14)70324-2 25662776 10.1016/S1474-4422(14)70324-2 PMC6742501

[4] Olney NT , Ong E, Goh SYM, et al Clinical and volumetric changes with increasing functional impairment in familial frontotemporal lobar degeneration. Alzheimers Dement. 2020;16(1):49–59. doi:10.1016/j.jalz.2019.08.196 31784375 10.1016/j.jalz.2019.08.196 PMC6988137

[5] Lee SE , Sias AC, Mandelli ML, et al Network degeneration and dysfunction in presymptomatic C9ORF72 expansion carriers. Neuroimage Clin. 2016;14:286–297. doi:10.1016/j.nicl.2016.12.006 28337409 10.1016/j.nicl.2016.12.006 PMC5349617

[6] Cash DM , Bocchetta M, Thomas DL, et al Patterns of gray matter atrophy in genetic frontotemporal dementia: Results from the GENFI study. Neurobiol Aging. 2018;62:191–196. doi:10.1016/j.neurobiolaging.2017.10.008 29172163 10.1016/j.neurobiolaging.2017.10.008 PMC5759893

[7] Bocchetta M , Todd EG, Peakman G, et al Differential early subcortical involvement in genetic FTD within the GENFI cohort. Neuroimage Clin. 2021;30:102646. doi:10.1016/j.nicl.2021.102646 33895632 10.1016/j.nicl.2021.102646 PMC8099608

[8] Cury C , Durrleman S, Cash DM, et al Spatiotemporal analysis for detection of pre-symptomatic shape changes in neurodegenerative diseases: Initial application to the GENFI cohort. Neuroimage. 2019;188:282–290. doi:10.1016/j.neuroimage.2018.11.063 30529631 10.1016/j.neuroimage.2018.11.063 PMC6414401

[9] Cardoso M J , Leung K, Modat M, et al STEPS: Similarity and truth estimation for propagated segmentations and its application to hippocampal segmentation and brain parcelation. Med Image Anal. 2013;17(6):671–684. doi:10.1016/j.media.2013.02.006 23510558 10.1016/j.media.2013.02.006

[10] Walhout R , Schmidt R, Westeneng HJ, et al Brain morphologic changes in asymptomatic C9orf72 repeat expansion carriers. Neurology. 2015;85(20):1780–1788. doi:10.1212/WNL.0000000000002135 26497991 10.1212/WNL.0000000000002135

[11] von Luxburg U . A tutorial on spectral clustering. arXiv:07110189 [cs], http://arxiv.org/abs/0711.0189, November 1 2007, preprint: not peer reviewed.

[12] Naqvi S , Sleyp Y, Hoskens H, et al Shared heritability of human face and brain shape. Nat Genet. 2021;53(6):830–839. doi:10.1038/s41588-021-00827-w 33821002 10.1038/s41588-021-00827-w PMC8232039

[13] Claes P , Roosenboom J, White JD, et al Genome-wide mapping of global-to-local genetic effects on human facial shape. Nat Genet 2018;50(3):414–423. doi:10.1038/s41588-018-0057-4 29459680 10.1038/s41588-018-0057-4 PMC5937280

[14] Gors D , Suetens P, Vandenberghe R, Claes P. Hierarchical spectral clustering of MRI for global-to-local shape analysis: Applied to brain variations in Alzheimer’s disease. IEEE 14th International Symposium on Biomedical Imaging (ISBI 2017); 2017:787–791. doi:10.1109/ISBI.2017.7950636

[15] Ashburner J . A fast diffeomorphic image registration algorithm. Neuroimage. 2007;38(1):95–113. doi:10.1016/j.neuroimage.2007.07.007 17761438 10.1016/j.neuroimage.2007.07.007

[16] Dahnke R , Ziegler G, Grosskreutz J, Gaser C. Retrospective quality assurance of MR images. 2013. doi:10.13140/RG.2.2.25494.91200

[17] Fowlkes C , Belongie S, Chung F, Malik J. Spectral grouping using the nystrom method. IEEE Trans Pattern Anal Machine Intell. 2004;26(2):214–225. doi:10.1109/TPAMI.2004.1262185 10.1109/TPAMI.2004.1262185 15376896

[18] Ayad HG , Kamel MS. Cumulative voting consensus method for partitions with variable number of clusters. IEEE Trans Pattern Anal Machine Intell. 2008;30(1):160–173. doi:10.1109/TPAMI.2007.1138 10.1109/TPAMI.2007.1138 18000332

[19] Ledesma R , Valero-Mora P. Determining the number of factors to retain in EFA: An easy-to-use computer program for carrying out parallel analysis. Published online 2007. doi:10.7275/WJNC-NM63

[20] Benjamini Y , Yekutieli D. The control of the false discovery rate in multiple testing under dependency. Ann Statist. 2001;29(4):1165–1188. doi:10.1214/aos/1013699998

[21] Gaser C , Kurth F. Manual Computational Anatomy Toolbox—CAT12. 2019. http://www.neuro.uni-jena.de/cat12/CAT12-Manual.pdf 10.1093/gigascience/giae049 PMC11299546 39102518

[22] Pernet CR . Robust Statistical Toolbox; 2020. Accessed 26 January 2022. https://github.com/CPernet/Robust_Statistical_Toolbox

[23] Rohrer JD , Ridgway GR, Modat M, et al Distinct profiles of brain atrophy in frontotemporal lobar degeneration caused by progranulin and tau mutations. Neuroimage. 2010;53(3):1070–1076. doi:10.1016/j.neuroimage.2009.12.088 20045477 10.1016/j.neuroimage.2009.12.088 PMC2941039

[24] Convery RS , Bocchetta M, Greaves CV, et al Abnormal pain perception is associated with thalamo-cortico-striatal atrophy in C9orf72 expansion carriers in the GENFI cohort. J Neurol Neurosurg Psychiatry. 2020;91(12):1325–1328. doi:10.1136/jnnp-2020-323279 32759310 10.1136/jnnp-2020-323279

[25] Lee SE , Khazenzon AM, Trujillo AJ, et al Altered network connectivity in frontotemporal dementia with C9orf72 hexanucleotide repeat expansion. Brain. 2014;137(11):3047–3060. doi:10.1093/brain/awu248 25273996 10.1093/brain/awu248 PMC4208465

[26] Franklin HD , Russell LL, Peakman G, et al The revised self-monitoring scale detects early impairment of social cognition in genetic frontotemporal dementia within the GENFI cohort. Alzheimers Res Ther. 2021;13(1):127. doi:10.1186/s13195-021-00865-w 34253227 10.1186/s13195-021-00865-w PMC8276486

[27] Bede P , Omer T, Finegan E, et al Connectivity-based characterisation of subcortical grey matter pathology in frontotemporal dementia and ALS: A multimodal neuroimaging study. Brain Imaging Behav. 2018;12(6):1696–1707. doi:10.1007/s11682-018-9837-9 29423814 10.1007/s11682-018-9837-9

[28] DeJesus-Hernandez M , Mackenzie IR, Boeve BF, et al Expanded GGGGCC hexanucleotide repeat in noncoding region of C9ORF72 causes chromosome 9p-linked FTD and ALS. Neuron. 2011;72(2):245–256. doi:10.1016/j.neuron.2011.09.011 21944778 10.1016/j.neuron.2011.09.011 PMC3202986

[29] De Vocht J , Blommaert J, Devrome M, et al Use of multimodal imaging and clinical biomarkers in presymptomatic carriers of C9orf72 repeat expansion. JAMA Neurol. 2020;77(8):1008–1017. doi:10.1001/jamaneurol.2020.1087 32421156 10.1001/jamaneurol.2020.1087 PMC7417970

[30] Bruffaerts R . Machine learning in neurology: What neurologists can learn from machines and vice versa. J Neurol. 2018;265:2745–2748. doi:10.1007/s00415-018-8990-9 30073503 10.1007/s00415-018-8990-9

[31] Staffaroni AM , Cobigo Y, Goh SYM, et al Individualized atrophy scores predict dementia onset in familial frontotemporal lobar degeneration. Alzheimers Dement. 2020;16(1):37–48. doi:10.1016/j.jalz.2019.04.007 31272932 10.1016/j.jalz.2019.04.007 PMC6938544

[32] Benjamini Y , Hochberg Y. Controlling the false discovery rate: A practical and powerful approach to multiple testing. J R Stat Soc Ser B Methodol. 1995;57(1):289–300. doi:10.1111/j.2517-6161.1995.tb02031.x

[33] Chen Q , Boeve BF, Senjem M, et al Rates of lobar atrophy in asymptomatic MAPT mutation carriers. Alzheimers Dement (N Y). 2019;5:338–346. doi:10.1016/j.trci.2019.05.010 31388560 10.1016/j.trci.2019.05.010 PMC6675939

[34] Chu SA , Flagan TM, Staffaroni AM, et al Brain volumetric deficits in MAPT mutation carriers: A multisite study. Ann Clin Transl Neurol. 2021;8(1):95–110. doi:10.1002/acn3.51249 33247623 10.1002/acn3.51249 PMC7818091

[35] Caroppo P , Le Ber I, Camuzat A, et al Extensive white matter involvement in patients with frontotemporal lobar degeneration: Think progranulin. JAMA Neurol. 2014;71(12):1562–1566. doi:10.1001/jamaneurol.2014.1316 25317628 10.1001/jamaneurol.2014.1316

[36] Sudre CH , Bocchetta M, Cash D, et al White matter hyperintensities are seen only in GRN mutation carriers in the GENFI cohort. Neuroimage Clin. 2017;15:171–180. doi:10.1016/j.nicl.2017.04.015 28529873 10.1016/j.nicl.2017.04.015 PMC5429247

[37] Sudre CH , Bocchetta M, Heller C, et al White matter hyperintensities in progranulin-associated frontotemporal dementia: A longitudinal GENFI study. Neuroimage Clin. 2019;24:102077. doi:10.1016/j.nicl.2019.102077 31835286 10.1016/j.nicl.2019.102077 PMC6911860

[38] Caponetti L , Castellano G, Corsini V. MR Brain image segmentation: A framework to compare different clustering techniques. Information. 2017;8(4):138. doi:10.3390/info8040138

[39] Gazzina S , Grassi M, Premi E, et al Education modulates brain maintenance in presymptomatic frontotemporal dementia. J Neurol Neurosurg Psychiatry. 2019;90(10):1124–1130. doi:10.1136/jnnp-2019-320439 31182509 10.1136/jnnp-2019-320439

