# Supplementary material for: Hierarchical spectral clustering reveals brain size and shape changes in asymptomatic carriers of C9orf72
Source: Brain Commun. 2022 Jul 18;4(4):fcac182. doi: 10.1093/braincomms/fcac182 (PMC9311825; doi:10.1093/braincomms/fcac182)
Supplement: fcac182_Supplementary_Data [file fcac182_supplementary_data.pdf]

Hierarchical spectral clustering reveals brain size and shape changes in asymptomatic carriers of *c9orf72* by Bruffaerts, Gors et al.:

Supplementary figures

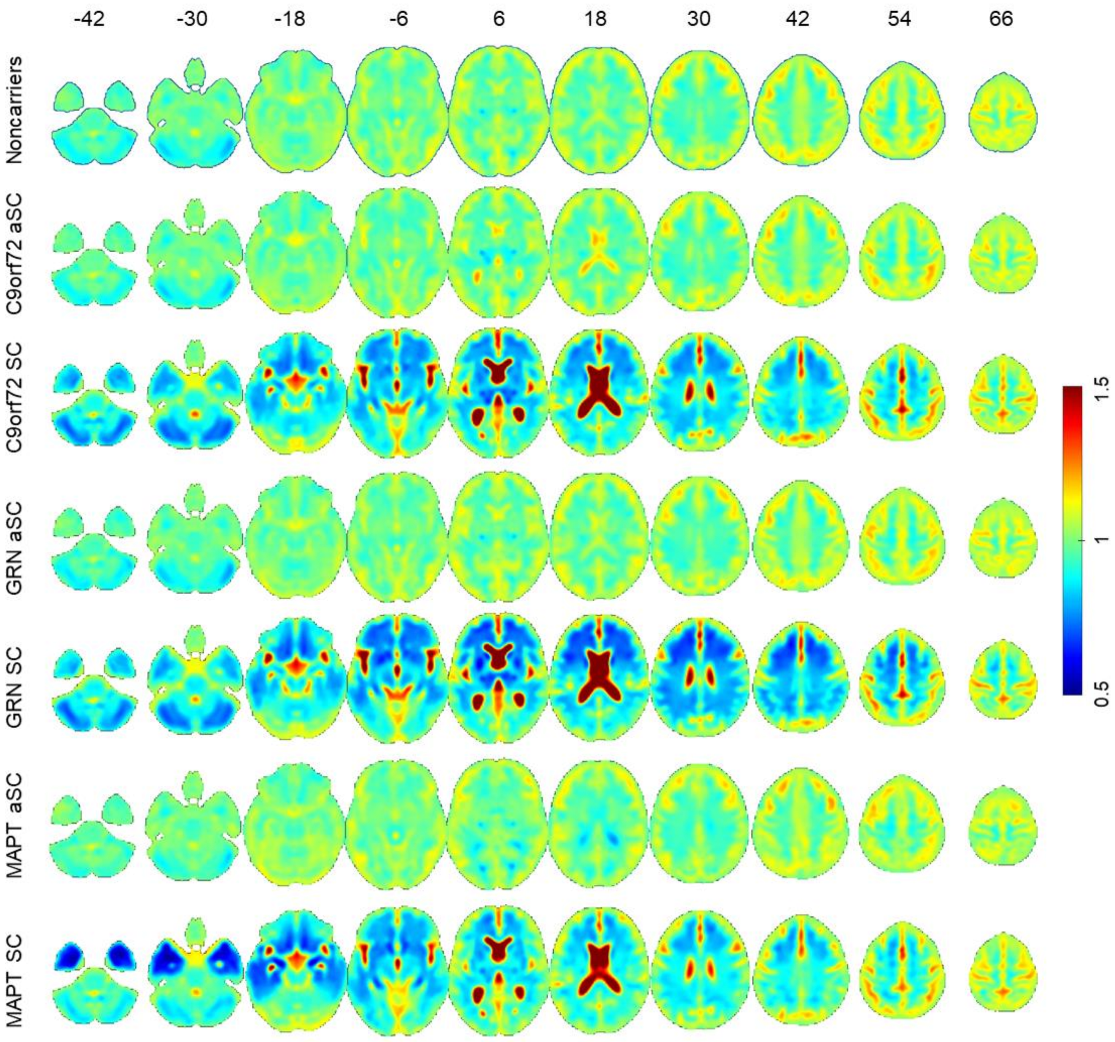

Supplementary Figure 1: Average Jacobian maps for noncarriers, C9orf72 asymptomatic carriers (aSC) and symptomatic carriers (SC), GRN aSC and SC, MAPT aSC and SC. Colorscale indicates the magnitude of the Jacobian (no units).

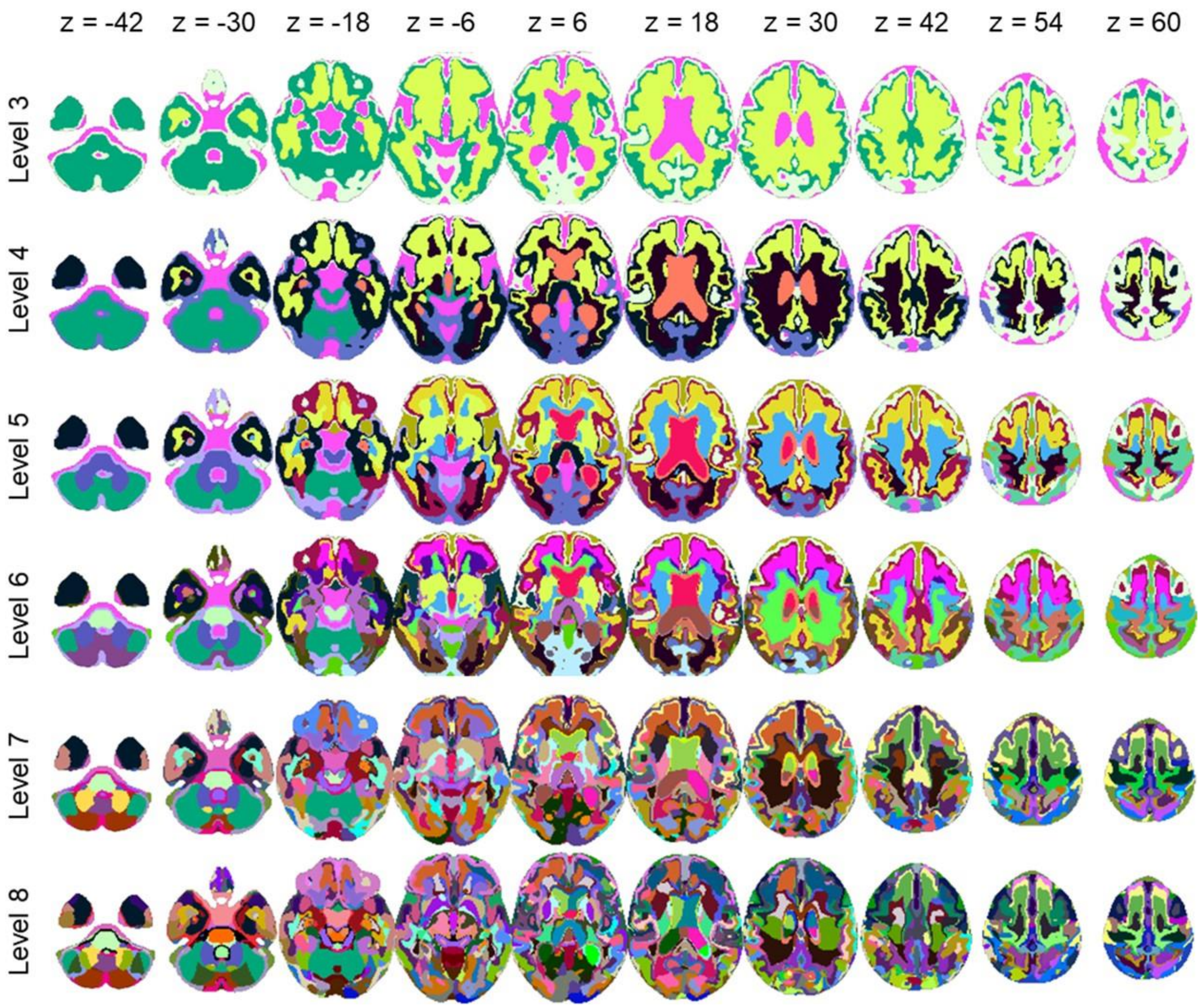

Supplementary Figure 2: Segmentation results: visualization of the 3*th* to 8*th* level of the hierarchical segmentation (random colorscale to ensure maximal contrast between adjacent segments).

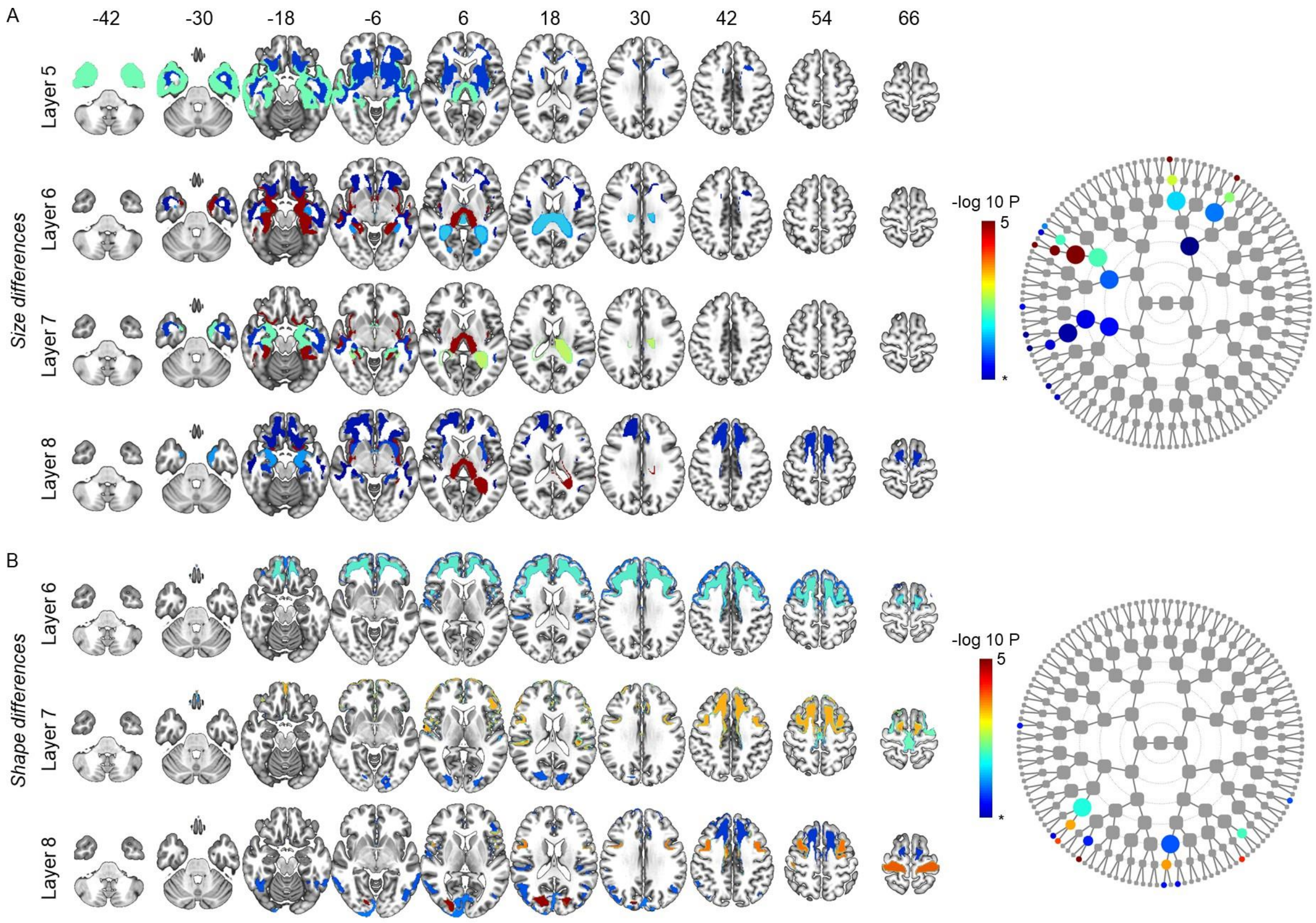

Supplementary Figure 3: Asymptomatic carriers C9orf72 versus noncarriers: additional global-to-local segment results for A) size and B) shape and their respective dendrograms. Asterisk indicates FDR adjusted significance (dep) p = 0.0007, -log p = 3.17, results below the FDR adjusted significance threshold are not illustrated). Nodes can be linked to their spatial coverage via Fig. 1A.

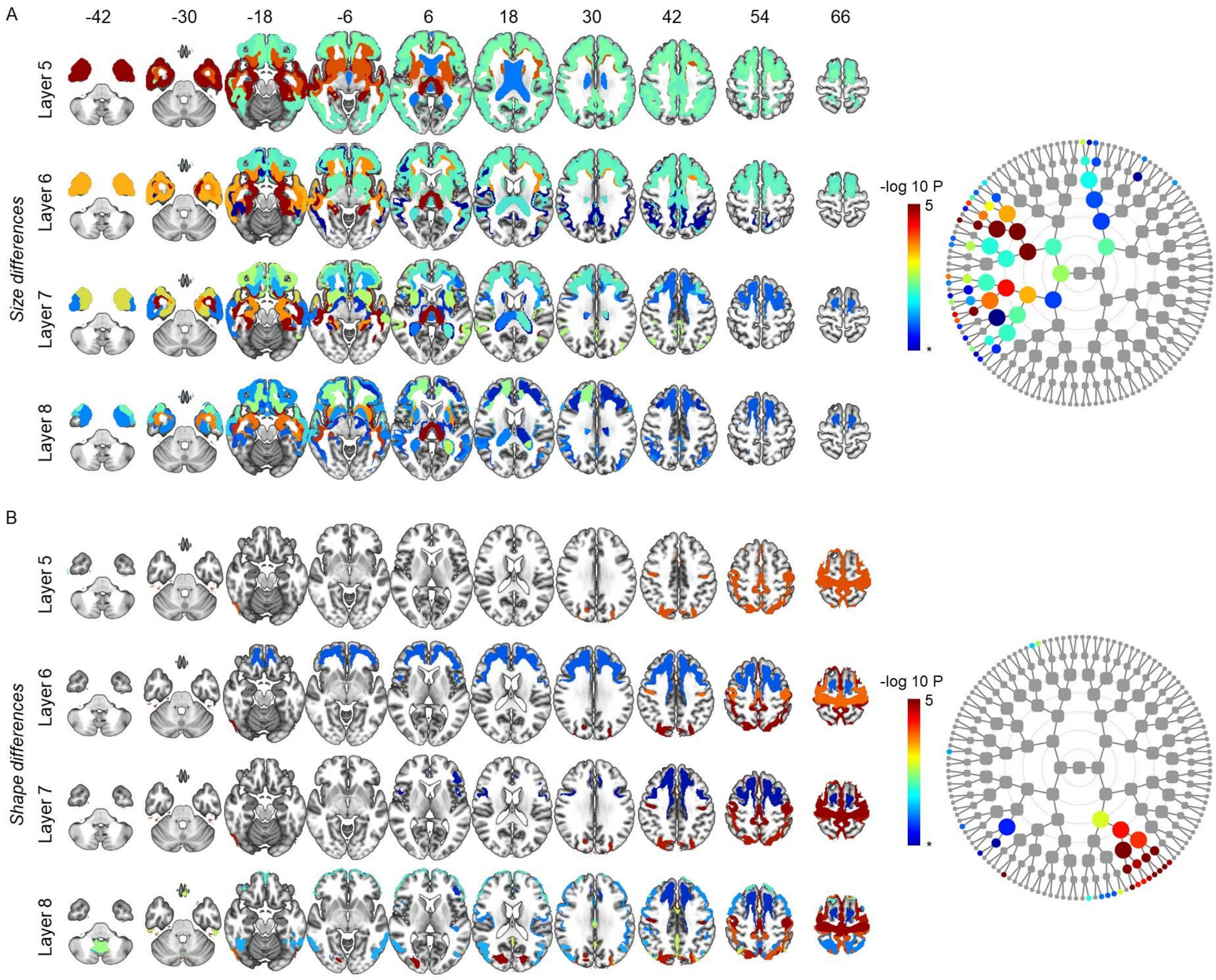

Supplementary Figure 4: Symptomatic carriers C9orf72 versus noncarriers: additional global-to-local segment results for A) size and B) shape and their respective dendrograms. Asterisk indicates FDR adjusted significance (dep) p = 0.0007, -log p = 3.17, results below the FDR adjusted significance threshold are not illustrated). Nodes can be linked to their spatial coverage via Fig. 1A.

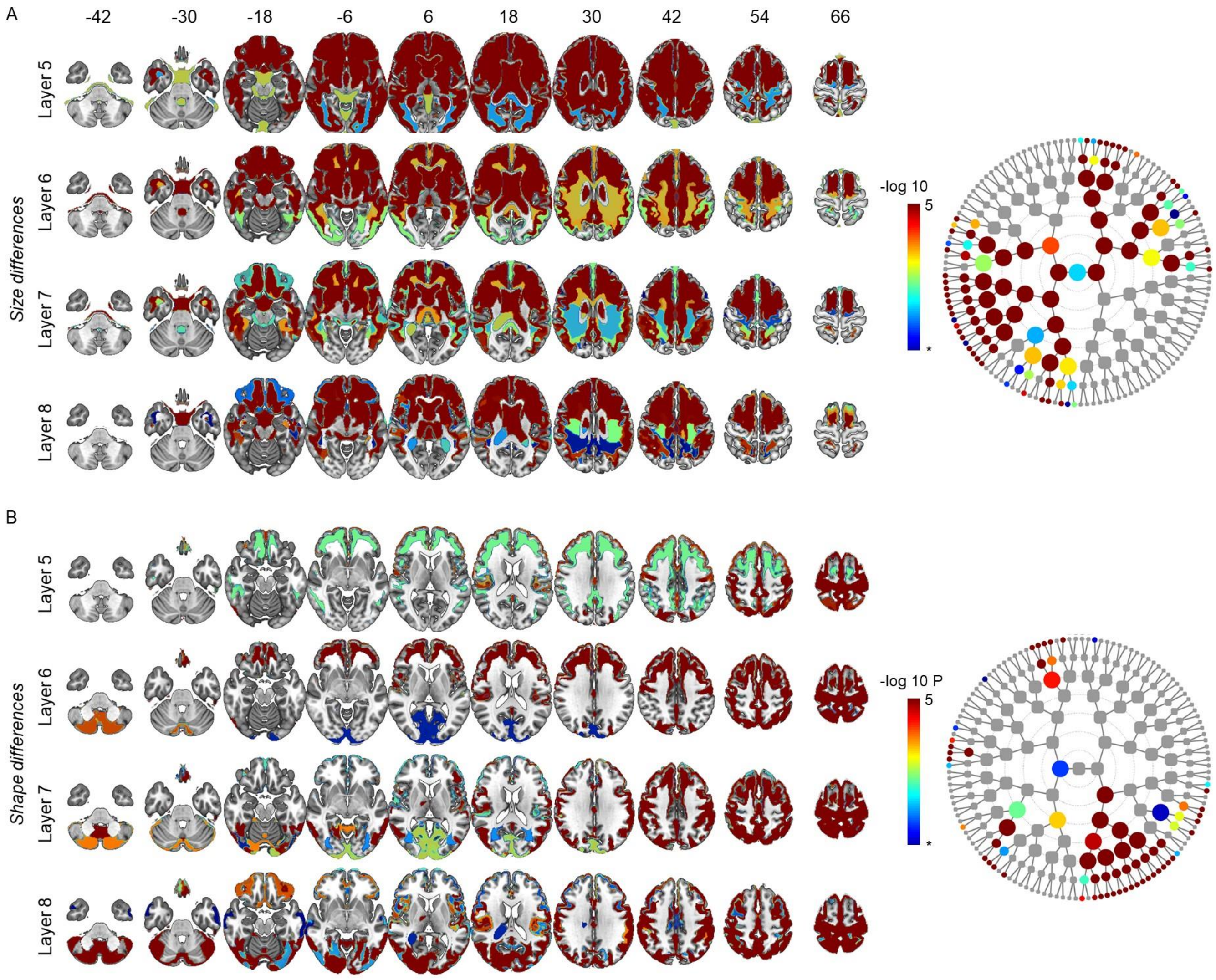

Supplementary Figure 5: Symptomatic carriers GRN versus noncarriers: additional global-to-local segment results for A) size and B) shape and their respective dendrograms. Asterisk indicates FDR adjusted significance (dep) p = 0.0007, -log p = 3.17, results below the FDR adjusted significance threshold are not illustrated). Nodes can be linked to their spatial coverage via Fig. 1A.

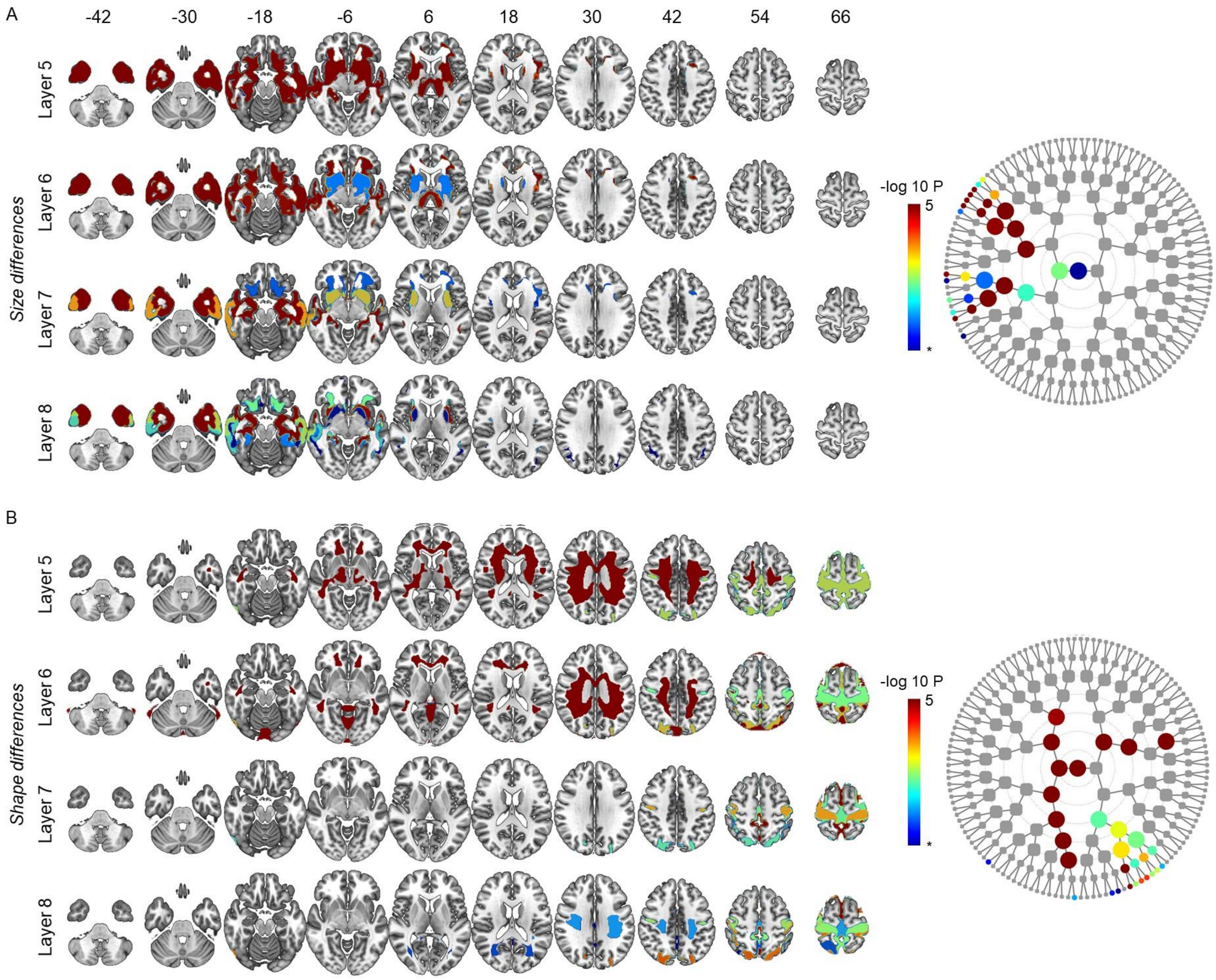

Supplementary Figure 6: Symptomatic carriers MAPT versus noncarriers: Global-to-local segment results for A) size and B) shape and their respective dendrograms. Asterisk indicates FDR adjusted significance (dep) p = 0.0007, -log p = 3.17, results below the FDR adjusted significance threshold are not illustrated). Nodes can be linked to their spatial coverage via Fig. 1A.

Supplementary files

The nifti images of the hierarchical spectral clustering segmentation results (levels 2 to 8) can be downloaded from the journal website.

GENFI consortium authors

Sónia Afonso[1], Maria Rosario Almeida[2], Sarah Anderl-Straub[3], Christin Andersson[4], Anna Antonell[5], Silvana Archetti[6], Andrea Arighi[7], Mircea Balasa[8], Myriam Barandiaran[9], Nuria Bargalló[10], Robart Bartha[11], Benjamin Bender[12], Alberto Benussi[13], Sandra Black[14], Martina Bocchetta[15], Sergi Borrego-Ecija[16], Jose Bras[17], Marta Canada[18], Valentina Cantoni[19], Paola Caroppo[20], David Cash[21], Miguel Castelo-Branco[22], Rhian Convery[23], Thomas Cope[24], Giuseppe Di Fede[25], Alina D´ıez[26] Diana Duro[27], Chiara Fenoglio[28], Catarina B. Ferreira[29], Nick Fox[30], Morris Freedman[31], Giorgio Fumagalli[32], Alazne Gabilondo[33], Roberto Gasparotti[34], Serge Gauthier[35], Stefano Gazzina[36], Giorgio Giaccone[37], Ana Gorostidi[38],Caroline Greaves[39], Rita Guerreiro[40], Carolin Heller[41], Tobias Hoegen[42], Begoña Indakoetxea[43], Vesna Jelic[44], Lize Jiskoot[45], Hans-Otto Karnath[46], Ron Keren[47], Tobias Langheinrich[48], Maria João Leitão[49], Albert Lladó[50], Sandra Loosli[51], Carolina Maruta[52], Simon Mead[53], Lieke Meeter[54], Gabriel Miltenberger[55], Rick van Minkelen[56], Sara Mitchell[57], Katrina Moore[58], Jennifer Nicholas[59], Linn Öijerstedt[60], Jaume Olives[61], Sebastien Ourselin[62], Alessandro Padovani[63], Jessica Panman[64], Janne M. Papma[65], Georgia Peakman[66], Yolande Pijnenburg[67], Enrico Premi[68], Sara Prioni[69], Catharina Prix[70], Rosa Rademakers[71],Veronica Redaelli[72], Tim Rittman[73], Ekaterina Rogaeva[74], Pedro Rosa-Neto[75], Giacomina Rossi[76], Martin Rossor[77], Beatriz Santiago[78], Elio Scarpini[79], Sonja Schönecker[80], Elisa Semler[81], Rachelle Shafei[82], Christen Shoesmith[83], Miguel Tábuas-Pereira[84], Mikel Tainta[85], Ricardo Taipa[86], David Tang-Wai[87], David L Thomas[88], Paul Thompson[89], Hakan Thonberg[90], Carolyn Timberlake[91], Pietro Tiraboschi[92], Emily Todd[93], Michele Veldsman[94], Ana Verdelho[95], Jorge Villanua[96], Jason Warren[97], Carlo Wilke[98], Ione Woollacott[99], Elisabeth Wlasich[100], Henrik Zetterberg[101], Miren Zulaica[102]

1. Instituto Ciencias Nucleares Aplicadas a Saude, Universidade de Coimbra, Coimbra, Portugal
2. Faculty of Medicine, University of Coimbra, Coimbra, Portugal
3. Department of Neurology, University of Ulm, Ulm, Germany
4. Department of Clinical Neuroscience, Karolinska Institutet, Stockholm, Sweden
5. Alzheimer's disease and Other Cognitive Disorders Unit, Neurology Service, Hospital Clinic, Barcelona,Spain
6. Biotechnology Laboratory, Department of Diagnostics, ASST Brescia Hospital, Brescia, Italy
7. Fondazione IRCCS Ca' Granda Ospedale Maggiore Policlinico, Neurodegenerative Diseases Unit,Milan, Italy; University of Milan, Centro Dino Ferrari, Milan, Italy
8. Alzheimer's disease and Other Cognitive Disorders Unit, Neurology Service, Hospital Clinic, Barcelona,Spain
9. Cognitive Disorders Unit, Department of Neurology, Donostia University Hospital, San Sebastian, Gipuzkoa, Spain; Neuroscience Area, Biodonostia Health Research Insitute, San Sebastian, Gipuzkoa, Spain
10. Imaging Diagnostic Center, Hospital Clinic, Barcelona, Spain
11. Department of Medical Biophysics, The University of Western Ontario, London, Ontario, Canada; Centre for Functional and Metabolic Mapping, Robarts Research Institute, The University of Western Ontario, London, Ontario, Canada
12. Department of Diagnostic and Interventional Neuroradiology, University of Tübingen,

Tübingen, Germany
13. Centre for Neurodegenerative Disorders, Department of Clinical and Experimental Sciences, University of Brescia, Italy
14. Sunnybrook Health Sciences Centre, Sunnybrook Research Institute, University of Toronto, Toronto, Canada
15. Department of Neurodegenerative Disease, Dementia Research Centre, UCL Institute of Neurology, Queen Square, London, UK
16. Alzheimer’s disease and Other Cognitive Disorders Unit, Neurology Service, Hospital Clinic, Barcelona, Spain
17. Center for Neurodegenerative Science, Van Andel Institute, Grand Rapids, Michigan, MI 49503,USA
18. CITA Alzheimer, San Sebastian, Gipuzkoa, Spain
19. Centre for Neurodegenerative Disorders, Neurology Unit, Department of Clinical and ExperimentalSciences, University of Brescia, Brescia, Italy
20. Fondazione IRCCS Istituto Neurologico Carlo Besta, Milano, Italy
21. Department of Neurodegenerative Disease, Dementia Research Centre, UCL Institute of Neurology,Queen Square, London, UK
22. Faculty of Medicine, University of Coimbra, Coimbra, Portugal
23. Department of Neurodegenerative Disease, Dementia Research Centre, UCL Institute of Neurology,Queen Square, London, UK
24. Department of Clinical Neuroscience, University of Cambridge, Cambridge, UK
25. Fondazione IRCCS Istituto Neurologico Carlo Besta, Milano, Italy
26. Neuroscience Area, Biodonostia Health Research Insitute, San Sebastian, Gipuzkoa, Spain
27. Faculty of Medicine, University of Coimbra, Coimbra, Portugal
28. Fondazione IRCCS Ca’ Granda Ospedale Maggiore Policlinico, Neurodegenerative Diseases Unit,Milan, Italy; University of Milan, Centro Dino Ferrari, Milan, Italy
29. Laboratory of Neurosciences, Institute of Molecular Medicine, Faculty of Medicine, University ofLisbon, Lisbon, Portugal
30. Department of Neurodegenerative Disease, Dementia Research Centre, UCL Institute of Neurology,Queen Square, London, UK
31. Baycrest Health Sciences, Rotman Research Institute, University of Toronto, Toronto, Canada
32. Fondazione IRCCS Ca’ Granda Ospedale Maggiore Policlinico, Neurodegenerative Diseases Unit,Milan, Italy; University of Milan, Centro Dino Ferrari, Milan, Italy
33. Neuroscience Area, Biodonostia Health Research Insitute, San Sebastian, Gipuzkoa, Spain
34. Neuroradiology Unit, University of Brescia, Brescia, Italy
35. Alzheimer Disease Research Unit, McGill Centre for Studies in Aging, Department of Neurology &Neurosurgery, McGill University, Montreal, Quebec, Canada
36. Neurology, ASST Brescia Hospital, Brescia, Italy
37. Fondazione IRCCS Istituto Neurologico Carlo Besta, Milano, Italy
38. Neuroscience Area, Biodonostia Health Research Insitute, San Sebastian, Gipuzkoa, Spain
39. Department of Neurodegenerative Disease, Dementia Research Centre, UCL Institute of Neurology,Queen Square, London, UK
40. Center for Neurodegenerative Science, Van Andel Institute, Grand Rapids, Michigan, MI 49503,USA
41. Department of Neurodegenerative Disease, Dementia Research Centre, UCL Institute of Neurology, Queen Square, London, UK
42. Neurologische Klinik, Ludwig-Maximilians-Universität München, Munich, Germany
43. Cognitive Disorders Unit, Department of Neurology, Donostia University Hospital, San Sebastian, Gipuzkoa, Spain; Neuroscience Area, Biodonostia Health Research Insitute, San Sebastian, Gipuzkoa, Spain
44. Division of Clinical Geriatrics, Karolinska Institutet, Stockholm, Sweden
45. Department of Neurology, Erasmus Medical Center, Rotterdam, Netherlands
46. Division of Neuropsychology, Hertie-Institute for Clinical Brain Research and Center of Neurology, University of Tubingen, Tubingen, Germany

47. The University Health Network, Toronto Rehabilitation Institute, Toronto, Canada
48. Division of Neuroscience and Experimental Psychology, Wolfson Molecular Imaging Centre, Univer- sity of Manchester, Manchester, UK
49. Centre of Neurosciences and Cell Biology, Universidade de Coimbra, Coimbra, Portugal
50. Alzheimer's disease and Other Cognitive Disorders Unit, Neurology Service, Hospital Clinic, Barcelona, Spain
51. Neurologische Klinik, Ludwig-Maximilians-Universität München, Munich, Germany
52. Laboratory of Language Research, Centro de Estudos Egas Moniz, Faculty of Medicine, University of Lisbon, Lisbon, Portugal
53. MRC Prion Unit, Department of Neurodegenerative Disease, UCL Institute of Neurology, Queen Square, London, UK
54. Department of Neurology, Erasmus Medical Center, Rotterdam, Netherlands
55. Faculty of Medicine, University of Lisbon, Lisbon, Portugal
56. Department of Clinical Genetics, Erasmus Medical Center, Rotterdam, Netherlands
57. Sunnybrook Health Sciences Centre, Sunnybrook Research Institute, University of Toronto, Toronto, Canada
58. Department of Neurodegenerative Disease, Dementia Research Centre, UCL Institute of Neurology, Queen Square, London, UK
59. Department of Medical Statistics, London School of Hygiene and Tropical Medicine, London, UK
60. Center for Alzheimer Research, Division of Neurogeriatrics, Department of Neurobiology, Care Sciences and Society, Bioclinicum, Karolinska Institutet, Solna, Sweden
61. Alzheimer's disease and Other Cognitive Disorders Unit, Neurology Service, Hospital Clinic, Barcelona, Spain
62. School of Biomedical Engineering & Imaging Sciences, King's College London, London, UK
63. Centre for Neurodegenerative Disorders, Department of Clinical and Experimental Sciences, Univer-sity of Brescia, Italy
64. Department of Neurology, Erasmus Medical Center, Rotterdam, Netherlands
65. Department of Neurology, Erasmus Medical Center, Rotterdam, Netherlands
66. Department of Neurodegenerative Disease, Dementia Research Centre, UCL Institute of Neurology, Queen Square, London, UK
67. Amsterdam University Medical Centre, Amsterdam VUmc, Amsterdam, Netherlands
68. Stroke Unit, ASST Brescia Hospital, Brescia, Italy
69. Fondazione IRCCS Istituto Neurologico Carlo Besta, Milano, Italy
70. Neurologische Klinik, Ludwig-Maximilians-Universität München, Munich, Germany
71. Department of Neurosciences, Mayo Clinic, Jacksonville, Florida, USA
72. Fondazione IRCCS Istituto Neurologico Carlo Besta, Milano, Italy
73. Department of Clinical Neurosciences, University of Cambridge, Cambridge, UK
74. Tanz Centre for Research in Neurodegenerative Diseases, University of Toronto, Toronto, Canada
75. Translational Neuroimaging Laboratory, McGill Centre for Studies in Aging, McGill University, Montreal, Québec, Canada
76. Fondazione IRCCS Istituto Neurologico Carlo Besta, Milano, Italy
77. Department of Neurodegenerative Disease, Dementia Research Centre, UCL Institute of Neurology, Queen Square, London, UK
78. Neurology Department, Centro Hospitalar e Universitario de Coimbra, Coimbra, Portugal
79. Fondazione IRCCS Ca' Granda Ospedale Maggiore Policlinico, Neurodegenerative Diseases Unit, Milan, Italy; University of Milan, Centro Dino Ferrari, Milan, Italy
80. Neurologische Klinik, Ludwig-Maximilians-Universität München, Munich, Germany
81. Department of Neurology, University of Ulm, Ulm
82. Department of Neurodegenerative Disease, Dementia Research Centre, UCL Institute of Neurology, Queen Square, London, UK
83. Department of Clinical Neurological Sciences, University of Western Ontario, London, Ontario, Canada

84. Neurology Department, Centro Hospitalar e Universitario de Coimbra, Coimbra, Portugal
85. Neuroscience Area, Biodonostia Health Research Insitute, San Sebastian, Gipuzkoa, Spain
86. Neuropathology Unit and Department of Neurology, Centro Hospitalar do Porto - Hospital de SantoAntonio, Oporto, Portugal
87. The University Health Network, Krembil Research Institute, Toronto, Canada
88. Neuroimaging Analysis Centre, Department of Brain Repair and Rehabilitation, UCL Institute ofNeurology, Queen Square, London, UK
89. Division of Neuroscience and Experimental Psychology, Wolfson Molecular Imaging Centre, Univer-sity of Manchester, Manchester, UK
90. Center for Alzheimer Research, Division of Neurogeriatrics, Karolinska Institutet, Stockholm, Sweden
91. Department of Clinical Neurosciences, University of Cambridge, Cambridge, UK
92. Fondazione IRCCS Istituto Neurologico Carlo Besta, Milano, Italy
93. Department of Neurodegenerative Disease, Dementia Research Centre, UCL Institute of Neurology,Queen Square, London, UK
94. Nuffield Department of Clinical Neurosciences, Medical Sciences Division, University of Oxford,Oxford, UK
95. Department of Neurosciences and Mental Health, Centro Hospitalar Lisboa Norte - Hospital de Santa Maria & Faculty of Medicine, University of Lisbon, Lisbon, Portugal
96. OSATEK, University of Donostia, San Sebastian, Gipuzkoa, Spain
97. Department of Neurodegenerative Disease, Dementia Research Centre, UCL Institute of Neurology, Queen Square, London, UK
98. Department of Neurodegenerative Diseases, Hertie-Institute for Clinical Brain Research and Center of Neurology, University of Tübingen, Tübingen, Germany; Center for Neurodegenerative Diseases (DZNE), Tübingen, Germany
99. Department of Neurodegenerative Disease, Dementia Research Centre, UCL Institute of Neurology,Queen Square, London, UK
100. Neurologische Klinik, Ludwig-Maximilians-Universität München, Munich, Germany
101. Dementia Research Institute, Department of Neurodegenerative Disease, UCL Institute of Neurology,Queen Square, London, UK
102. Neuroscience Area, Biodonostia Health Research Insitute, San Sebastian, Gipuzkoa, Spain
